# Multiscale Structure–Property Relationships in High-Viscosity Modified Asphalt with Different Polymer Contents

**DOI:** 10.3390/ma19214549

**Published:** 2026-10-28

**Authors:** Shuolei Huang, Yihui Xie, Mingjun Hu, Jianping Zhu, Zhengqi Zhang, Zichao Zhang

**Affiliations:** 1 Key Laboratory for Special Area Highway Engineering of Ministry of Education, Chang’an University, Xi’an 710064, China; 2 Liaoning Transportation Research Institute Co., Ltd., Shenyang 110015, China; 3 School of Infrastructure Engineering, Dalian University of Technology, Dalian 116024, China; xyh@mail.dlut.edu.cn (Y.X.); 17201529975@mail.dlut.edu.cn (Z.Z.); 4 Xinjiang Key Laboratory of Green Construction and Smart Traffic Control of Transportation Infrastructure, Urumqi 830017, China

**Keywords:** high-viscosity modified asphalt, molecular weight distribution, component composition, rheological properties, grey relational analysis

## Abstract

High-viscosity modified asphalt (HVMA) is widely used in porous and high-performance asphalt pavements because of its excellent high-temperature stability and elastic recovery. However, the relationships among modifier dosage, microscopic characteristics, and rheological performance remain insufficiently understood. In this study, HVMAs with different high-viscosity modifier dosages were characterized using conventional physical property tests, Fourier transform infrared spectroscopy, gel permeation chromatography, frequency and temperature sweeps, rheological master curves, and multiple stress creep recovery tests. Grey relational analysis was further conducted to evaluate multiscale structure–property associations. The results showed that increasing modifier dosage decreased penetration and non-recoverable creep compliance, while increasing softening point, low-temperature ductility, rotational viscosity, complex modulus, percent recovery, and viscoelastic transition temperature. Both 12% HVMA and 16% HVMA met the technical requirements for porous asphalt pavements, while 12% HVMA provided a more balanced compromise between performance and viscosity within the investigated range. Increasing modifier dosage also increased the weight-average molecular weight, polydispersity index, and polymer- and asphaltene-related molecular-weight fractions, while decreasing the maltene-related fraction. These changes may reflect the redistribution of asphalt components associated with polymer modification and the contribution of polymer-related species within overlapping apparent molecular-weight ranges. HVMA exhibited clear temperature- and frequency-dependent rheological responses. Under low-temperature and high-frequency conditions, the response was more strongly associated with the asphalt matrix, whereas the polymer-related elastic contribution became increasingly evident under high-temperature and low-frequency conditions, as reflected by the development of a phase-angle plateau. Grey relational analysis showed that the viscoelastic transition temperature had relatively high relational grades with SBS-related structural indicators, asphaltene-related characteristics, and molecular-weight parameters. Percent recovery and the phase-angle plateau were more strongly associated with polymer-related indicators, whereas non-recoverable creep compliance and complex modulus at low and intermediate temperatures showed stronger associations with maltene-related characteristics. At high temperatures, the complex modulus exhibited relatively high relational grades with both polymer- and asphalt-related parameters. These findings provide a basis for understanding dosage-dependent structure–property relationships and for selecting appropriate modifier contents in HVMA.

## 1. Introduction

With the development of urban transportation infrastructure toward greener, more functional, and longer-lasting systems, functional pavement structures such as porous asphalt pavements, open-graded friction courses, drainage and noise-reducing pavements, and thin asphalt overlays have been widely applied [1]. These pavement structures generally feature high interconnected air-void contents and relatively thin asphalt films, making them susceptible to raveling, aggregate loss, rutting, and cracking under the combined effects of traffic loading, moisture intrusion, temperature fluctuations, and oxidative aging [2,3]. Therefore, the asphalt binders used in such applications are required to exhibit high adhesion, thermal stability, elastic recovery, and resistance to permanent deformation. High-viscosity modified asphalt (HVMA), characterized by high viscosity, softening point, and elastic recovery, can enhance asphalt–aggregate adhesion and improve the structural stability of asphalt mixtures [4,5]. It has therefore become a critical binder for porous asphalt pavements and other high-performance asphalt pavement systems.

HVMA is commonly prepared by incorporating a relatively high dosage of a polymer-based high-viscosity modifier into base asphalt. Previous studies [6] have suggested that the polymer components in high-viscosity modifiers can absorb light fractions from asphalt and undergo swelling, followed by the development of polymer-rich structures through molecular-chain entanglement and physical interactions. This process substantially alters the component composition, colloidal structure, and viscoelasticity of the asphalt binder. Compared with conventional SBS-modified asphalt, HVMA generally exhibits higher high-temperature viscosity and greater elastic recovery. However, excessive polymer content may also increase construction viscosity and consequently hinder mixing and compaction. Therefore, determining an appropriate modifier dosage and clarifying its effects on the microscopic structure and macroscopic performance of HVMA are essential for performance optimization and engineering application.

In recent years, extensive studies have been conducted on the conventional physical properties, rheological behavior, storage stability, aging resistance, and mixture performance of HVMA [7,8]. Previous findings generally indicate that high-viscosity modifiers increase the softening point, viscosity, complex modulus, and elastic recovery of asphalt while reducing its non-recoverable creep compliance, thereby improving its high-temperature rutting resistance and resistance to permanent deformation [9]. Frequency sweep, temperature sweep, and rheological master-curve analyses have further shown that polymer-modified asphalt often exhibits a phase angle plateau in the intermediate-to-low frequency or intermediate-to-high temperature range [10,11]. This phenomenon is generally attributed to the elastic contribution of the spatial polymer network at elevated temperatures. In addition, Fourier transform infrared spectroscopy, fluorescence microscopy, atomic force microscopy, and gel permeation chromatography have been employed to characterize polymer chain-segment structure, phase morphology, and molecular weight distribution, thereby providing important evidence for interpreting the modification mechanisms of HVMA [12,13]. However, these techniques have mostly been applied separately, and quantitative relationships between dosage-dependent molecular/compositional characteristics and rheological responses remain insufficiently established.

Nevertheless, existing studies have largely focused on either macroscopic performance or individual microscopic characteristics, while quantitative relationships between dosage-dependent molecular/compositional changes and rheological responses remain insufficiently understood [14]. On the one hand, the incorporation of a high-viscosity modifier not only increases the polymer content of the system but also alters the relative proportions and molecular weight distributions of the asphaltene and maltene fractions through the absorption of light components, polymer swelling, and possible polymer degradation [15]. Polymer-network reinforcement and asphalt colloidal-structure reconstruction often occur simultaneously, making it difficult to distinguish their respective contributions to macroscopic performance. On the other hand, the rheological behavior of HVMA is strongly dependent on temperature and frequency. The relative contributions of the asphalt and polymer phases under low-temperature and high-frequency conditions may differ substantially from those under high-temperature and low-frequency conditions. However, systematic evidence regarding the temperature- and frequency-dependent changes in the relative rheological contributions of the asphalt matrix and polymer-related structures, as well as their relationship with the phase-angle plateau, remains limited [16]. Moreover, different microscopic indicators may exert different degrees of influence on rheological parameters such as the viscoelastic transition temperature, complex modulus, percent recovery, and non-recoverable creep compliance, whereas conventional qualitative analyses are insufficient to identify the key microscopic factors governing each performance indicator.

The objective of this study is to clarify how high-viscosity modifier dosage affects the molecular/compositional characteristics and rheological behavior of HVMA, and to establish their multiscale structure–property relationships. It is hypothesized that dosage-induced changes in polymer-related structure and molecular-weight distribution are associated with corresponding changes in temperature- and frequency-dependent rheological responses [17]. First, penetration, softening point, low-temperature ductility, and rotational viscosity tests were conducted to evaluate the conventional physical properties of HVMA. Fourier transform infrared spectroscopy and gel permeation chromatography were then employed to characterize the polymer chain-segment features, molecular weight distribution, and variations in the polymer, asphaltene, and maltene fractions. Peak deconvolution of the GPC curves was further performed to compare the peak distributions and area fractions of the different components. Subsequently, frequency sweep, temperature sweep, rheological master-curve, and multiple stress creep recovery tests were conducted to investigate the evolution of complex modulus, phase angle, storage modulus, loss modulus, viscoelastic transition behavior, elastic recovery, and resistance to permanent deformation. Particular attention was given to elucidating the stage-dependent dominance of the asphalt and polymer phases over different temperature and frequency ranges. Finally, grey relational analysis was employed to quantify the relationships between chemical composition, molecular weight distribution, component composition, and rheological indicators, thereby identifying the key microscopic factors governing different macroscopic properties [18]. The novelty of this study lies in integrating molecular/compositional characterization with rheological analysis across three modifier dosages (8%, 12%, and 16%), and further using grey relational analysis to quantitatively evaluate the associations between microscopic characteristics and macroscopic rheological responses.

## 2. Materials and Methods

### 2.1. Materials

The base asphalt used in this study was Sinopec 70# neat asphalt (70PEN) (Sinopec Corp., Beijing, China), while the styrene–butadiene–styrene (SBS) modified asphalt (SBSMA) was prepared using an I-D type SBS modifier (Baling Petrochemical Co., Ltd., Yueyang, China) with a dosage of 4.5%. The high-viscosity modifier adopted in this study was commercially available high-viscosity polymer particles (Sobute New Materials Co., Ltd., Nanjing, China), and its basic properties have been reported in previous studies [19]. Three high-viscosity modified asphalt (HVMA) with polymer contents of 8%, 12%, and 16% were prepared and denoted as 8% HVMA, 12% HVMA, and 16% HVMA, respectively.

The preparation procedure was as follows: the base asphalt was first heated to 150 °C until a molten state was achieved, while the high-viscosity polymer particles were preheated at 100 °C. The preheated particles were then incorporated into the asphalt matrix, followed by heating to 180 °C and mechanical stirring at 250 r/min for 30 min to promote sufficient particle swelling. Subsequently, high-speed shearing was applied at 180 °C and 4000 r/min for 60 min to ensure uniform dispersion of the swollen particles within the asphalt binder. Finally, the mixture was conditioned at 180 °C under 300 r/min stirring for 30 min to complete the development process, yielding the prepared HVMAs.

### 2.2. Macroscopic Performance Tests

#### 2.2.1. Basic Physical Tests

The basic physical properties of asphalt binders, including penetration, softening point, and ductility, were determined in accordance with the corresponding procedures specified in T0604, T0605, and T0606 of JTG E20-2011. The penetration and softening point tests were conducted at 25 °C. For the ductility measurement, the testing temperature was set at 15 °C for the BA, whereas a lower temperature of 5 °C was adopted for the HVMA. Penetration, softening point, ductility, and rotational viscosity tests were each performed in triplicate, and the average values were used for analysis.

#### 2.2.2. Frequency Sweep Test

Dynamic shear rheometer (DSR) (MCR 302, Anton Paar, Graz, Austria) was employed to characterize the rheological response of HVMAs through frequency sweep tests over a frequency range of 0.1–30 Hz. The measurements were performed at seven temperatures, including 15, 25, 35, 45, 55, 65, and 75 °C. Before each measurement, the asphalt samples were conditioned at the target temperature for 10 min to ensure uniform thermal equilibrium throughout the specimen. Based on our previous studies [20,21,22] on high-viscosity/polymer-modified asphalt, strain amplitudes of 0.15% and 1.5% were selected for tests conducted at 15–25 °C and 35–75 °C, respectively, to minimize potential nonlinear viscoelastic effects. During the frequency sweep process, the complex modulus |G^*^| and phase angle (*δ*) of the HVMAs were recorded. All DSR measurements were performed using two independent replicate specimens, and the average values were reported.

#### 2.2.3. Temperature Sweep Test

Temperature sweep tests were performed on HVMAs using a dynamic shear rheometer (DSR) over a temperature range of 15–75 °C. The measurements were conducted at a constant frequency of 10 Hz, with strain amplitudes of 1.5% and 0.15% applied in the high-temperature region (25–75 °C) and low-temperature region (15–25 °C), respectively, to ensure that the testing conditions remained within the linear viscoelastic (LVE) range of the binders. During the temperature sweep process, the complex modulus (|G^*^|) and phase angle (*δ*) were continuously recorded to evaluate the temperature-dependent viscoelastic behavior of the HVMAs. All DSR measurements were performed using two independent replicate specimens, and the average values were reported.

#### 2.2.4. Master Curve Construction 

To extend the effective frequency domain for rheological characterization, time–temperature superposition was applied to generate the rheological master curves of HVMAs at a reference temperature of 45 °C. The complex modulus master curves were fitted using the sigmoid function, while the corresponding phase angle master curves were described by the double-logistic (DL) model [23,24,25]. The temperature shift factors were optimized through nonlinear least-squares regression, and identical shift factors were used for the complex modulus and phase angle master curves. The fitting quality was evaluated using the coefficient of determination (*R*^2^ > 0.9).

(1) $$\log\left|G^{*}\right|=\delta +\frac{\alpha }{1+e^{\left(\beta +\gamma \left(\log f_{r}\right)\right)}}$$

(2) $$\delta =\delta_{P}-\delta_{P}\cdot H\left(f_{r}-f_{P}\right)\cdot \left(1-e^{-S_{R}{\left(\log\left(f_{r}/f_{P}\right)\right)}^{2}}\right)+\delta_{L}\cdot H\left(f_{P}-f_{r}\right)\cdot \left(1-e^{-S_{L}{\left(\log\left(f_{P}/f_{r}\right)\right)}^{2}}\right)$$

For Equation (1), *f*_r_ represents the reduced frequency (Hz). The parameter *δ* denotes the lower asymptote, while *α* corresponds to the difference between the upper and lower asymptotes. The parameters *β* and *γ* are shape-related coefficients, where *β* determines the horizontal position of the transition region of the curve, and *γ* governs the slope of the intermediate-frequency asymptotic region.

For Equation (2), *δ* represents the phase angle (°), and *f*_r_ denotes the reduced frequency (Hz). *f*_p_ is the characteristic frequency corresponding to the onset of the plateau region of the phase angle curve (Hz), and *δ*_p_ represents the phase angle in the plateau region (°). *S*_R_, *S*_L_, and *δ*_L_ are shape parameters. Specifically, *S*_R_ controls the approaching rate toward the high-frequency asymptote, *S*_L_ determines the approaching rate toward the low-frequency asymptote, and *δ*_L_ describes the magnitude of phase angle variation in the low-frequency region. *H*(*f*_r_ − *f*_p_)and *H*(*f*_p_ − *f*_r_) represent the Heaviside step functions.

#### 2.2.5. Viscoelastic Transition Temperature (VTT) Determination

Based on the measured complex modulus (|G^*^|) and phase angle (*δ*) obtained from the temperature sweep tests, the storage modulus (*G*′) and loss modulus (*G*″) of the HVMAs were calculated to further characterize their temperature-dependent viscoelastic characteristics. At relatively low temperatures, *G*′ remained higher than *G*″, indicating that the binders exhibited predominantly elastic behavior due to the restricted molecular mobility. With increasing temperature, *G*″ gradually exceeded *G*′, suggesting a transition toward a more viscous-dominated response caused by enhanced molecular movement. The viscoelastic transition temperature (VTT) was identified as the temperature corresponding to the intersection of the *G*′ and *G*″ curves, where the elastic and viscous components contributed equally to the overall rheological response.

#### 2.2.6. Multiple Stress Creep Recovery (MSCR) Test Procedure

The multiple stress creep recovery (MSCR) test was conducted to evaluate the elastic recovery capability and high-temperature permanent deformation resistance of HVMAs. According to AASHTO TP70-1 [26], the MSCR procedure consisted of two sequential stress levels. Initially, 20 creep-recovery cycles were applied under a stress level of 0.1 kPa, followed by 10 cycles under an elevated stress level of 3.2 kPa. Each cycle included a 1 s loading period and a subsequent 9 s recovery period. The average percent recovery (*R*_0.1_ and *R*_3.2_) and non-recoverable creep compliance (*J*_nr,0.1_ and *J*_nr,3.2_) were subsequently calculated using Equations (3) and (4) [27]. The calculation was based on the 11th–20th cycles at 0.1 kPa and the 1st–10th cycles at 3.2 kPa, respectively, to minimize the influence of initial loading instability and obtain representative MSCR parameters. The MSCR test was performed using two independent replicate specimens.

(3) $$R=\frac{\varepsilon_{p}-\varepsilon_{u}}{\varepsilon_{p}}\times 100\%$$

(4) $$J_{\mathrm{nr}}=\frac{\varepsilon_{u}}{\sigma }$$

In Equations (3) and (4), *R*_0.1_ and *R*_3.2_ represent the percent strain recovery (%) of HVMAs under creep stress levels of 0.1 kPa and 3.2 kPa, respectively. *ε*_p_ denotes the peak strain, while *ε*_u_ represents the unrecovered strain after the recovery period. *σ* is the applied creep stress. *J*_nr,0.1_ and *J*_nr,3.2_ refer to the non-recoverable creep compliance (kPa^−1^) measured at creep stress levels of 0.1 kPa and 3.2 kPa, respectively [28].

### 2.3. Microscopic Performance Tests

#### 2.3.1. Attenuated Total Reflectance Fourier Transform Infrared Spectroscopy (ATR-FTIR) Test

Fourier transform infrared spectroscopy (FTIR) coupled with an attenuated total reflectance (ATR) accessory was employed to characterize the chemical functional groups of asphalt binders and polymers [29]. In this study, a Bruker TENSOR FTIR spectrometer (Bruker Optik GmbH, Ettlingen, Germany) equipped with a diamond ATR crystal was used to analyze the chemical characteristics of HVMAs. The spectra were collected over a wavenumber range of 4000–600 cm^−1^ with 32 scans for each measurement.

Approximately 1–2 g of asphalt sample was uniformly spread onto the ATR diamond crystal and compressed using the upper clamp before testing. For polymer identification, the characteristic absorption peaks of polybutadiene (PB) and polystyrene (PS) located at approximately 966 cm^−1^ and 699 cm^−1^, respectively, were analyzed. Each sample was measured three times independently, and the average peak area was used for analysis.

#### 2.3.2. Gel Permeation Chromatography (GPC) Test

Gel permeation chromatography (GPC) was employed to characterize the molecular weight distribution of HVMAs. The GPC analysis was performed using a Waters 1515 high-performance liquid chromatography system equipped with a Waters 2414 refractive index detector (Waters Corporation, Milford, MA, USA). Polystyrene standards (Shodex^®^ series) with known molecular weights were first used to establish the calibration curve between elution time and molecular weight. Subsequently, HVMA samples were dissolved in tetrahydrofuran (THF) (Sinopharm Chemical Reagent Co., Ltd., Shanghai, China) at a concentration of 2–3 mg/mL and injected into the chromatographic system at a flow rate of 1 mL/min after filtration.

The molecular weight distribution curves were obtained by converting the RI signals using the calibration curve. The characteristic regions in the GPC profiles were assigned to polymer-, asphaltene-, and maltene-related molecular-weight fractions according to their elution times. These fractions represent operational classifications based on apparent molecular weight rather than chemically isolated components. The peak position, intensity, and width were analyzed to evaluate the molecular weight distribution and compositional variations of HVMAs [30]. Considering the relatively high cost of GPC testing and the good stability of the analytical method, only one independent measurement was performed for each asphalt sample. The repeatability and statistical uncertainty of the GPC results warrant further investigation in future studies.

### 2.4. Grey Relational Analysis

In this study, grey relational analysis (GRA) was employed to establish the correlations between the macroscopic performance of HVMAs and their microscopic compositions and structural characteristics. The fundamental concept of GRA is to evaluate the degree of association between variables by quantifying the similarity of the geometric patterns of their variation curves. A higher similarity in the changing trends of two variable sequences indicates a stronger relational degree, whereas a lower similarity corresponds to a weaker relationship. Compared with other statistical analysis approaches, such as regression analysis, analysis of variance (ANOVA), and principal component analysis (PCA), GRA exhibits distinct advantages for engineering problems involving limited or incomplete data availability. Although GRA does not impose strict requirements on sample size or data distribution, the resulting relational grades may still be influenced by the normalization method, distinguishing coefficient, and the number of available data levels.

The procedures of grey relational analysis are described as follows:

(1) Determination of reference and comparison sequences

The original reference sequence and comparison sequences are defined as *X*_0_ and *X*_i_, respectively, where *i* = 1, 2, …, *n*.

(2) Initialization of data sequences

To eliminate the influence of different dimensions among variables, the original reference and comparison sequences were normalized using the mean-value normalization method. The transformed sequences are denoted as *Y*_0_ and *Y*_i_, respectively, which can be expressed as:

(5) $$x_{0}^{\left(0\right)}\left(t\right)=\left\{x_{0}^{\left(0\right)}\left(1\right),x_{0}^{\left(0\right)}\left(2\right),\ldots ,x_{0}^{\left(0\right)}\left(m\right)\right\}=\left\{\frac{x_{0}(1)}{\sum_{t=1}^{m}x_{0}\left(t\right)/m},\frac{x_{0}(2)}{\sum_{t=1}^{m}x_{0}\left(t\right)/m},\ldots ,\frac{x_{0}(m)}{\sum_{t=1}^{m}x_{0}\left(t\right)/m}\right\}$$

(6) $$x_{i}^{\left(0\right)}\left(t\right)=\left\{x_{i}^{\left(0\right)}\left(1\right),x_{i}^{\left(0\right)}\left(2\right),\ldots ,x_{i}^{\left(0\right)}\left(m\right)\right\}=\left\{\frac{x_{i}(1)}{\sum_{t=1}^{m}x_{i}\left(t\right)/m},\frac{x_{i}(2)}{\sum_{t=1}^{m}x_{i}\left(t\right)/m},\ldots ,\frac{x_{i}(m)}{\sum_{t=1}^{m}x_{i}\left(t\right)/m}\right\}$$

(3) Calculation of grey relational coefficients

The grey relational coefficient between the reference sequence and each comparison sequence was calculated according to Equation (7):

(7) $$Y_{0i}\left(t\right)=\frac{\Delta_{\min}+W\Delta_{\max}}{\left|x_{0}(t)-x_{i}(t)\right|+W\Delta_{\max}}$$

where *Y*_0i_(*t*) represents the grey relational coefficient at point *t*; *W* is the distinguishing coefficient within the range of 0–1, which is commonly assigned as 0.5; *Δ*_min_ and *Δ*_max_ represent the minimum and maximum differences between the reference sequence and comparison sequences, respectively.

(4) Determination of grey relational grade

The grey relational grade between the reference sequence and each comparison sequence was obtained using Equation (8):

(8) $$V_{0i}=\frac{1}{m}\sum_{i=1}^{m}Y_{0i}\left(t\right)$$

where *Y*_0i_ denotes the grey relational grade, and *Y*_0i_(*t*) represents the grey relational coefficient.

## 3. Results and Discussion

### 3.1. Microscopic Characteristics of HVMA with Different Polymer Contents

#### 3.1.1. Characteristic Functional Groups

**Figure 1:**
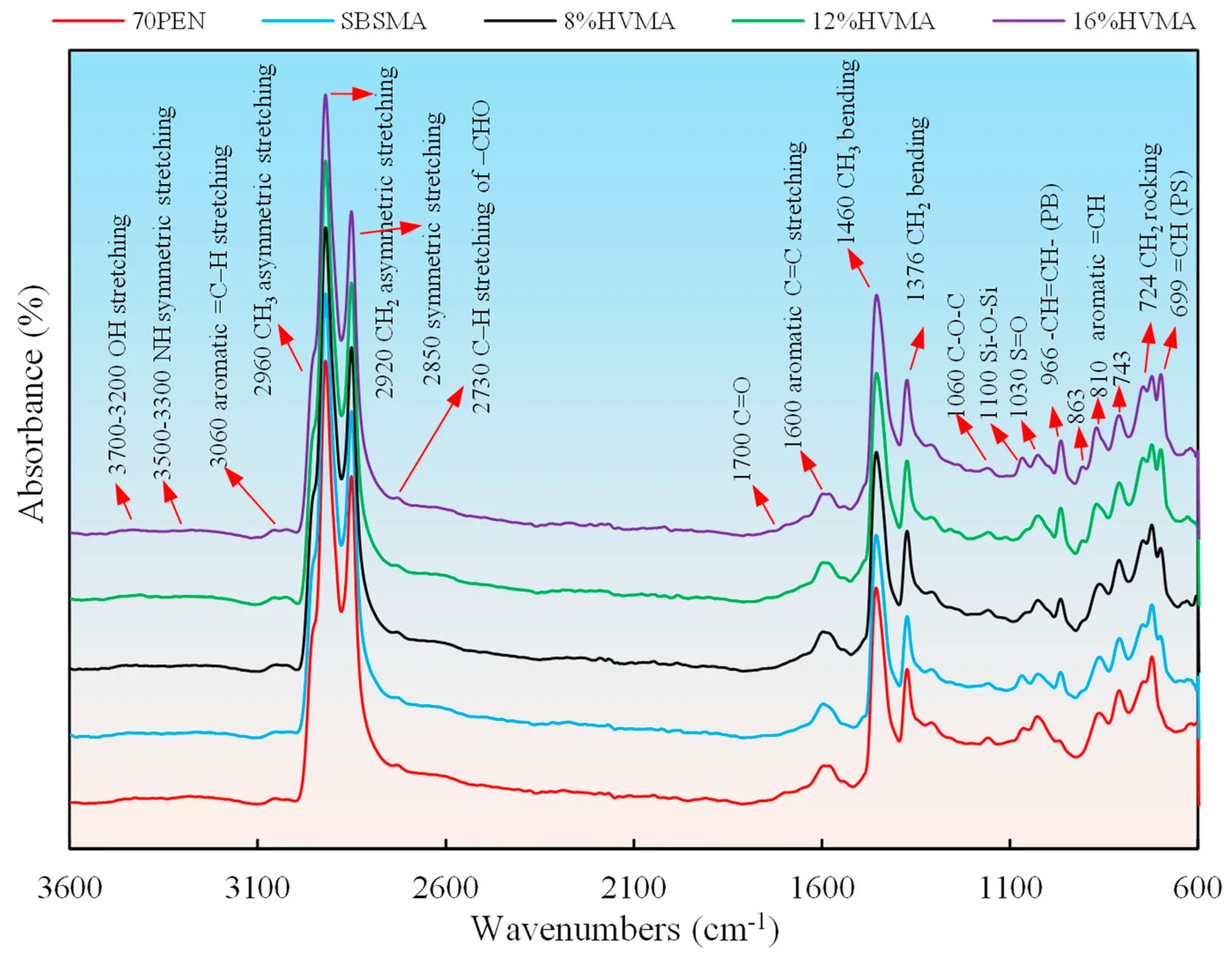
Chemical functional group results of HVMA.

The chemical functional groups of 70PEN, SBSMA, and HVMA were analyzed by FTIR spectroscopy, and the corresponding spectra are presented in Figure 1. As shown in Figure 1, 70PEN, SBSMA, and HVMA exhibited several common characteristic absorption bands, indicating the presence of similar chemical structures among these asphalt binders. The identified absorption peaks, arranged in descending wavenumber order, were assigned as follows: 3700–3200 cm^−1^ corresponding to O–H stretching vibration, 3500–3300 cm^−1^ attributed to N–H symmetric stretching vibration, 2960 cm^−1^ associated with asymmetric stretching vibration of CH_3_ groups, 2920 and 2850 cm^−1^ assigned to asymmetric and symmetric stretching vibrations of CH_2_ groups, 1700 cm^−1^ related to C=O stretching vibration, 1600 cm^−1^ attributed to aromatic C=C stretching vibration, 1460 cm^−1^ corresponding to CH_3_ bending vibration, 1376 cm^−1^ assigned to CH_2_ bending vibration, 1030 cm^−1^ related to S=O stretching vibration, 863, 810, and 743 cm^−1^ associated with aromatic =C–H out-of-plane deformation vibrations, and 724 cm^−1^ corresponding to in-plane rocking vibration of CH_2_ groups.

In addition, SBSMA and HVMA exhibited two additional characteristic absorption bands associated with polymer structures, including the band at 966 cm^−1^ attributed to the out-of-plane deformation of –CH=CH– groups in polybutadiene (PB) segments and the band at 699 cm^−1^ assigned to the out-of-plane deformation of substituted aromatic C–H groups in polystyrene (PS) segments [29]. Therefore, the integrated peak areas of the PB characteristic band at 966 cm^−1^ and the PS characteristic band at 699 cm^−1^ were selected as indicators for evaluating the polymer content and polymer structural characteristics in HVMAs [31].

**Figure 2:**
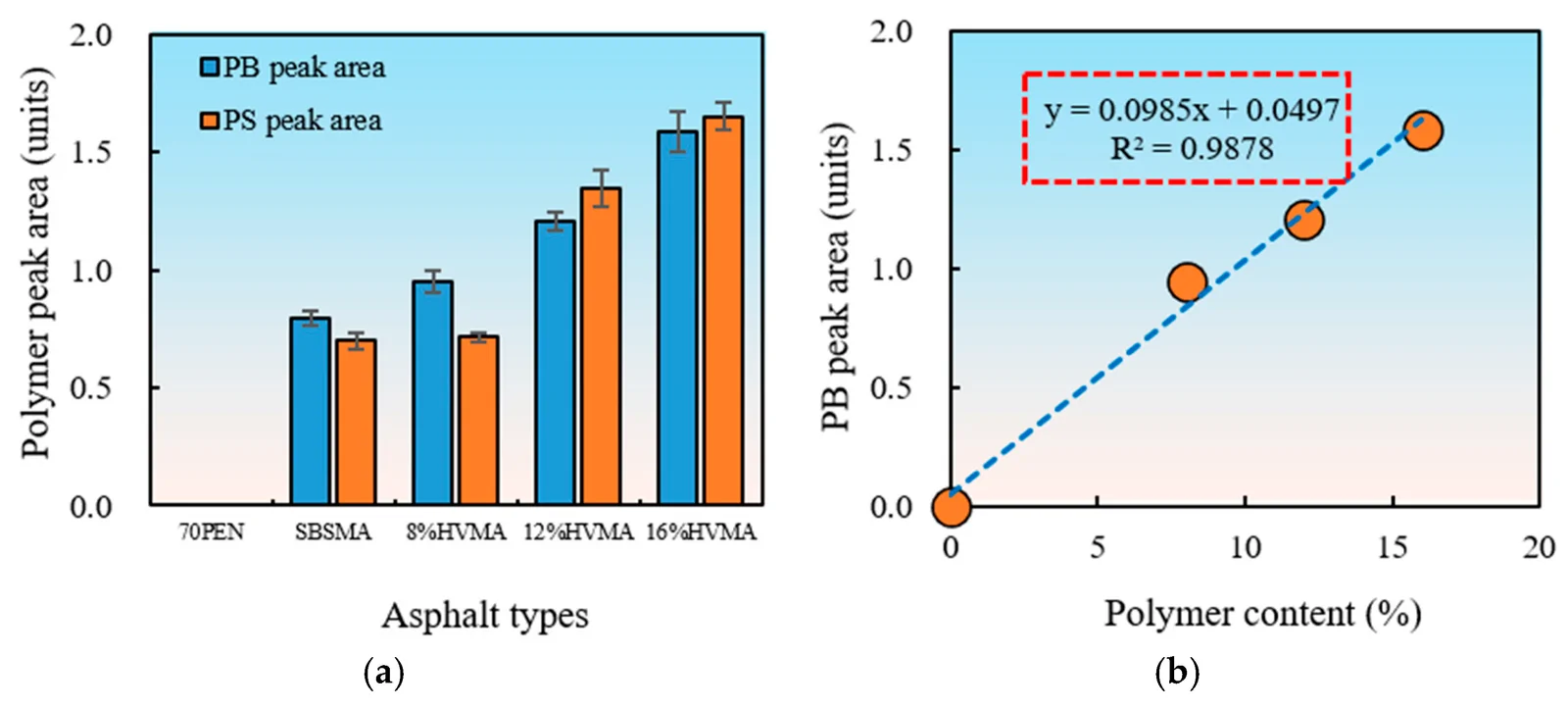
Polymer peak areas and normalized polymer content curves of HVMA: (**a**) Polymer peak area; (**b**) normalized polymer curve. Error bars represent standard deviation (SD), *n* = 3. The red dashed square in (**b**) highlights the linear fitting equation and the coefficient of determination (*R^2^*).

The integrated peak areas of PB and PS characteristic bands in SBSMA and HVMA with different polymer contents are presented in Figure 2a [32]. As shown in Figure 2a, the PB and PS peak areas of 8% HVMA were comparable to those of SBSMA, indicating that the effective polymer content in 8% HVMA was approximately equivalent to that in 4.5% SBSMA. With increasing high-viscosity modifier content, both PB and PS peak areas of HVMA gradually increased, suggesting an enhanced polymer-related structure within the asphalt binder.

The PB segment in SBS is more susceptible to degradation than the PS segment due to the presence of α-hydrogen atoms adjacent to C=C bonds and unsaturated double bonds, which results in lower chemical stability under environmental aging conditions [33]. The degradation of PB segments can directly disrupt the three-dimensional polymer network structure of SBS, leading to the deterioration of the polymer-modified asphalt phase structure. Therefore, the PB characteristic band is considered more suitable than the PS band for evaluating the effective polymer content in polymer-modified asphalt binders.

Based on the relationship between PB peak area and high-viscosity modifier content, a normalized calibration curve was established for HVMA, as shown in Figure 2b. The results demonstrate a strong linear correlation between the PB peak area and modifier content, with a coefficient of determination (*R*^2^) of 0.98. This calibration curve provides a quantitative approach for evaluating the retained effective polymer content of HVMA during long-term aging.

#### 3.1.2. Molecular Weight Distribution

**Figure 3:**
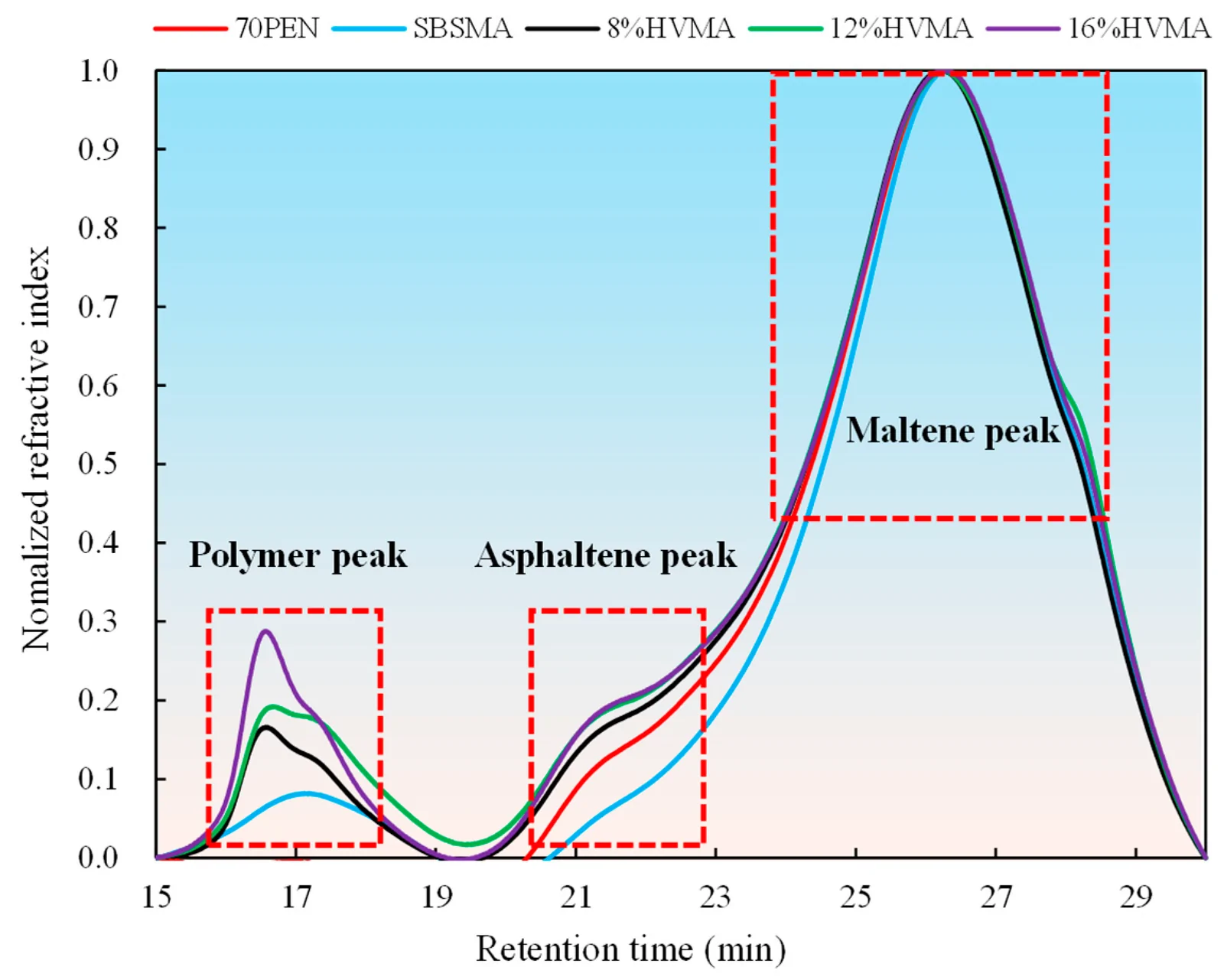
Molecular weight distribution curves of HVMA.

The molecular weight distribution curves of HVMA obtained from GPC analysis are presented in Figure 3. As shown in Figure 3, the 70PEN binder exhibited two characteristic peaks with elution times of 21 and 26.5 min, corresponding to the asphaltene and maltene fractions, respectively. In contrast, SBSMA and HVMA displayed three distinct peaks located at approximately 16.5, 21, and 26.5 min, which were assigned to the polymer, asphaltene, and maltene fractions, respectively [24,34]. Furthermore, the polymer peak intensity of SBSMA was pronouncedly lower than that of HVMA, indicating a lower polymer-related molecular component in SBSMA. With increasing high-viscosity modifier content, the intensities of both polymer and asphaltene peaks in HVMA gradually increased, suggesting an increase in the polymer content and asphaltene fraction within the HVMA system [35].

The calculated number-average molecular weight (*M*_n_), weight-average molecular weight (*M*_w_), and polydispersity index (PDI) values of HVMA are presented in Table 1. *M*_w_ is more sensitive to variations in high-molecular-weight components, whereas *M*_n_ is more responsive to changes in low-molecular-weight fractions. The polydispersity index (PDI) represents the breadth of the molecular weight distribution. The *M*_w_ of HVMA is markedly higher than those of SBSMA and 70PEN, and it further increases with increasing polymer content. This phenomenon is mainly attributed to the higher proportion of high-molecular-weight polymer components in HVMA. In contrast to *M*_w_, the variations in *M*_n_ among different asphalt binders are relatively minor. Furthermore, HVMA exhibits a substantially higher PDI value than SBSMA and 70PEN, indicating a much broader molecular weight distribution. Among the three binders, SBSMA shows an intermediate molecular weight distribution range, while 70PEN exhibits the narrowest distribution. A similar broadening of molecular-weight distribution after polymer modification has also been reported by Zhou et al. [36], who attributed the rheological enhancement of high-viscosity asphalt to changes in polymer–asphalt interactions and network structure.

**Figure 4:**
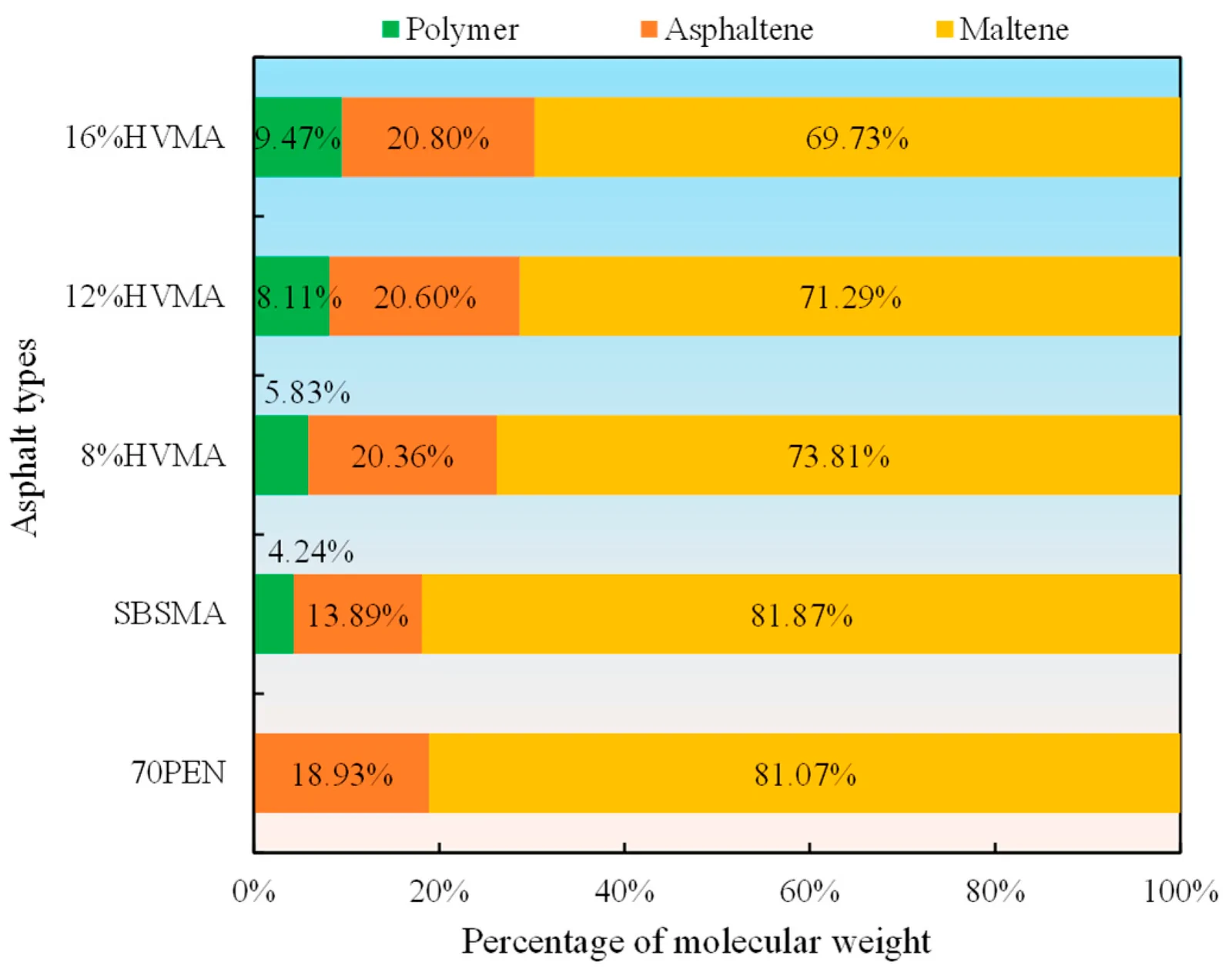
Percentage contents of different components in HVMA.

According to the study by Daly et al. [37], HVMA was divided into three fractions based on molecular weight: polymer components (molecular weight > 19,000), asphaltene components (molecular weight of 3000–19,000), and maltene components (molecular weight < 3000), as shown in Figure 4. As illustrated in Figure 4, HVMA exhibits a higher asphaltene-related molecular-weight fraction and a lower maltene-related fraction than 70PEN. With increasing HVM dosage, the polymer- and asphaltene-related fractions increase, whereas the maltene-related fraction decreases. It should be emphasized that the increase in the asphaltene-related fraction does not necessarily represent an actual increase in chemically defined asphaltenes, because polymer species within the same apparent molecular-weight range may also contribute to this fraction. These results further indicate that the incorporation of high-viscosity modifiers promotes the absorption of light components from asphalt, leading to polymer swelling. Consequently, the maltene fraction decreases, while the asphaltene fraction increases in the modified asphalt.

**Figure 5:**
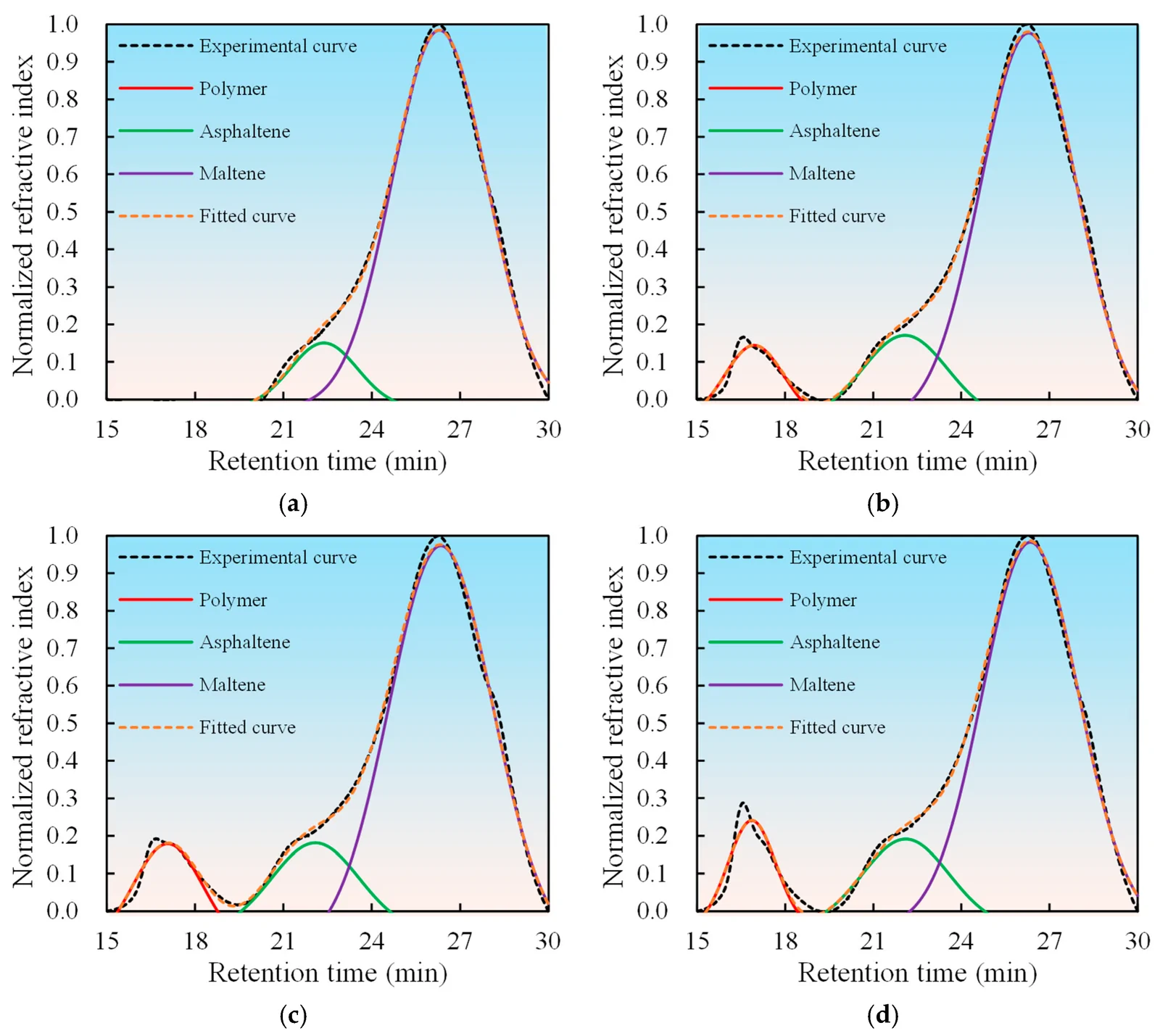
Deconvolution fitting results of the molecular weight distribution curve of HVMA: (**a**) 70PEN; (**b**) 8% HVMA; (**c**) 12% HVMA; (**d**) 16% HVMA.

The GPC curves of 70PEN and HVMA were subjected to peak deconvolution analysis to compare the molecular weight distributions and relative proportions of the polymer, asphaltene, and maltene fractions. The fitting results are presented in Figure 5. Peak deconvolution was performed using Origin software (OriginLab Corporation, Northampton, MA, USA). The characteristic elution-time ranges of the polymer-, asphaltene-, and maltene-related fractions were used to define the initial peak positions, and multiple Gaussian functions were adopted to fit the experimental GPC curves. The peak position, width, and intensity were optimized using nonlinear least-squares fitting. The goodness of fit was evaluated using the coefficient of determination (*R*^2^), which quantifies the agreement between the experimental GPC curves and the reconstructed curves, with *R*^2^ > 0.85 adopted as the fitting acceptance criterion.

As shown in Figure 5, the characteristic peak of the polymer fraction was mainly distributed within an elution time range of 15–18.5 min, whereas the asphaltene fraction exhibited a major peak distribution between 19.5 and 24.5 min. The maltene fraction showed characteristic peaks primarily located within 22.5–30 min. The elution time ranges of the characteristic peaks for different fractions partially overlapped, which may introduce uncertainties when distinguishing different components solely based on molecular weight classification. Consequently, the molecular weight-based method for separating asphalt fractions may have certain limitations in accurately identifying the individual components. Because the deconvoluted area fractions may be affected by the initial peak settings and peak overlap, the resulting percentages are interpreted primarily in terms of relative dosage-dependent trends rather than absolute component contents.

**Figure 6:**
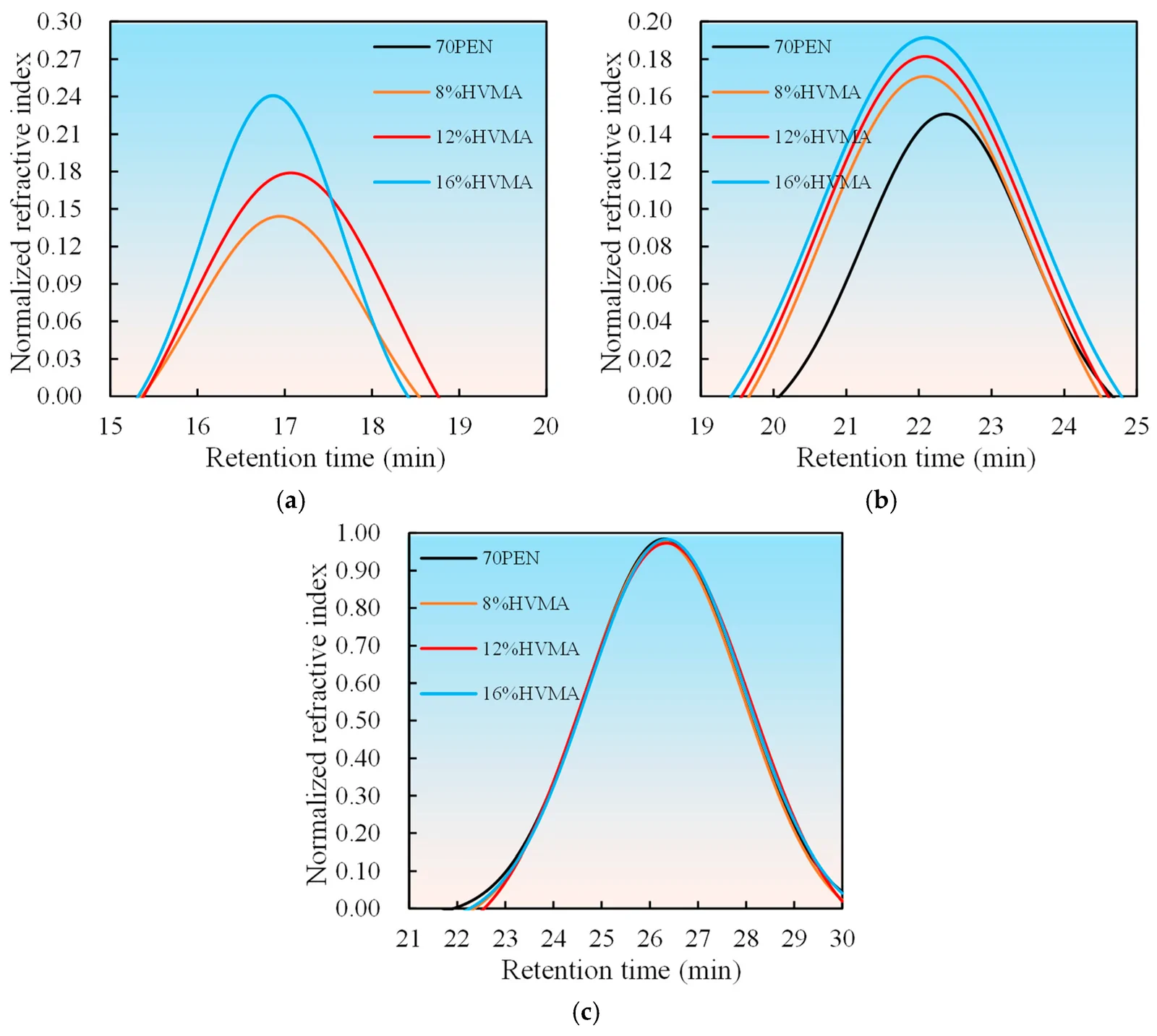
Comparative analysis of HVMA component contents: (**a**) Polymer; (**b**) asphaltene; (**c**) maltene.

Figure 6 presents a comparative analysis of the distributions of the polymer, asphaltene, and maltene peaks in 70PEN and HVMA with different high-viscosity modifier (HVM) contents. With increasing HVM dosage, the height of the polymer peak gradually increases, accompanied by a slight shift toward shorter elution times. For 8% HVMA, the polymer peak is located within an elution time range of 15.37–18.52 min, corresponding to a molecular weight range of 38,022–708,570. For 12% HVMA, the polymer peak exhibits an elution time range of 15.37–18.75 min, corresponding to a molecular weight range of 33,042–708,570. For 16% HVMA, the polymer peak is distributed within 15.32–18.40 min, corresponding to a molecular weight range of 41,036–756,457. These results indicate that increasing the high-viscosity modifier content increases the amount of polymer components in HVMA but has a minimal effect on the molecular weight distribution range of the polymer fraction.

With increasing high-viscosity modifier content, the height of the asphaltene peak in HVMA gradually increases, and its distribution range becomes broader, particularly shifting toward shorter elution times (higher molecular weights). The asphaltene peak of 70PEN is located within an elution time range of 20.09–24.66 min, corresponding to a molecular weight range of 2490–11,780. For 8% HVMA, the asphaltene peak appears within 19.67–24.79 min, corresponding to a molecular weight range of 2322–20,158. For 12% HVMA, the asphaltene peak is distributed within 19.57–24.59 min, corresponding to a molecular weight range of 2583–21,172. For 16% HVMA, the asphaltene peak exhibits an elution time range of 19.42–24.78 min, corresponding to a molecular weight range of 2335–22,835. These findings demonstrate that the incorporation of high-viscosity modifiers increases both the content and molecular weight of the asphaltene fraction in HVMA. The observed increase in the asphaltene-related molecular-weight fraction may be associated with the absorption of light asphalt components during polymer swelling, as suggested in previous studies [38]. In addition, low-molecular-weight polymer chains or degraded polymer fragments may also contribute to signals within this molecular-weight range. These mechanisms are therefore regarded as literature-supported interpretations rather than direct evidence from the present GPC measurements. As shown in Figure 6c, no marked differences are observed in either the peak height or distribution range of the maltene fraction between 70PEN and HVMA. The molecular weight of the maltene fraction is mainly distributed within the range of 0–8000. This indicates that the incorporation of high-viscosity modifiers has a minimal influence on the molecular weight distribution of the maltene fraction in asphalt [39].

Based on the relative areas enclosed by the fitted component curves after peak deconvolution, the proportions of polymer, asphaltene, and maltene fractions in HVMA were further calculated, as presented in Table 2. With increasing high-viscosity modifier content, the polymer and asphaltene fractions in HVMA increase, whereas the maltene fraction decreases. This trend is consistent with the variation in component contents determined by the molecular weight classification method described above. However, discrepancies exist between the component contents obtained by peak deconvolution and those calculated based on molecular weight classification. The difference in the polymer fraction determined by the two methods is relatively minor, whereas the calculated contents of the asphaltene and maltene fractions show considerable differences. The molecular weight classification method is relatively simple and has been widely used for quantifying asphalt components. However, this method cannot effectively account for the overlapping regions among the polymer, asphaltene, and maltene peaks. In contrast, the peak deconvolution method can more accurately separate and quantify the overlapping regions among these three fractions. Nevertheless, this approach is more complicated, and additional errors may be introduced during the fitting process. Therefore, the selection of an appropriate method for component quantification should consider the balance between analytical accuracy and operational complexity.

It should be noted that GPC primarily reflects apparent molecular-weight distributions rather than direct chemical separation of asphalt and polymer species. Therefore, the polymer-, asphaltene-, and maltene-related fractions should be regarded as operational molecular-weight classifications with partially overlapping ranges. Changes in these fractions cannot directly confirm polymer swelling, degradation, or chemical conversion, and the proposed mechanisms are interpreted mainly as literature-supported explanations of the observed dosage-dependent trends.

### 3.2. Macroscopic Performance of HVMA with Different Polymer Contents

#### 3.2.1. Basic Physical Properties

**Figure 7:**
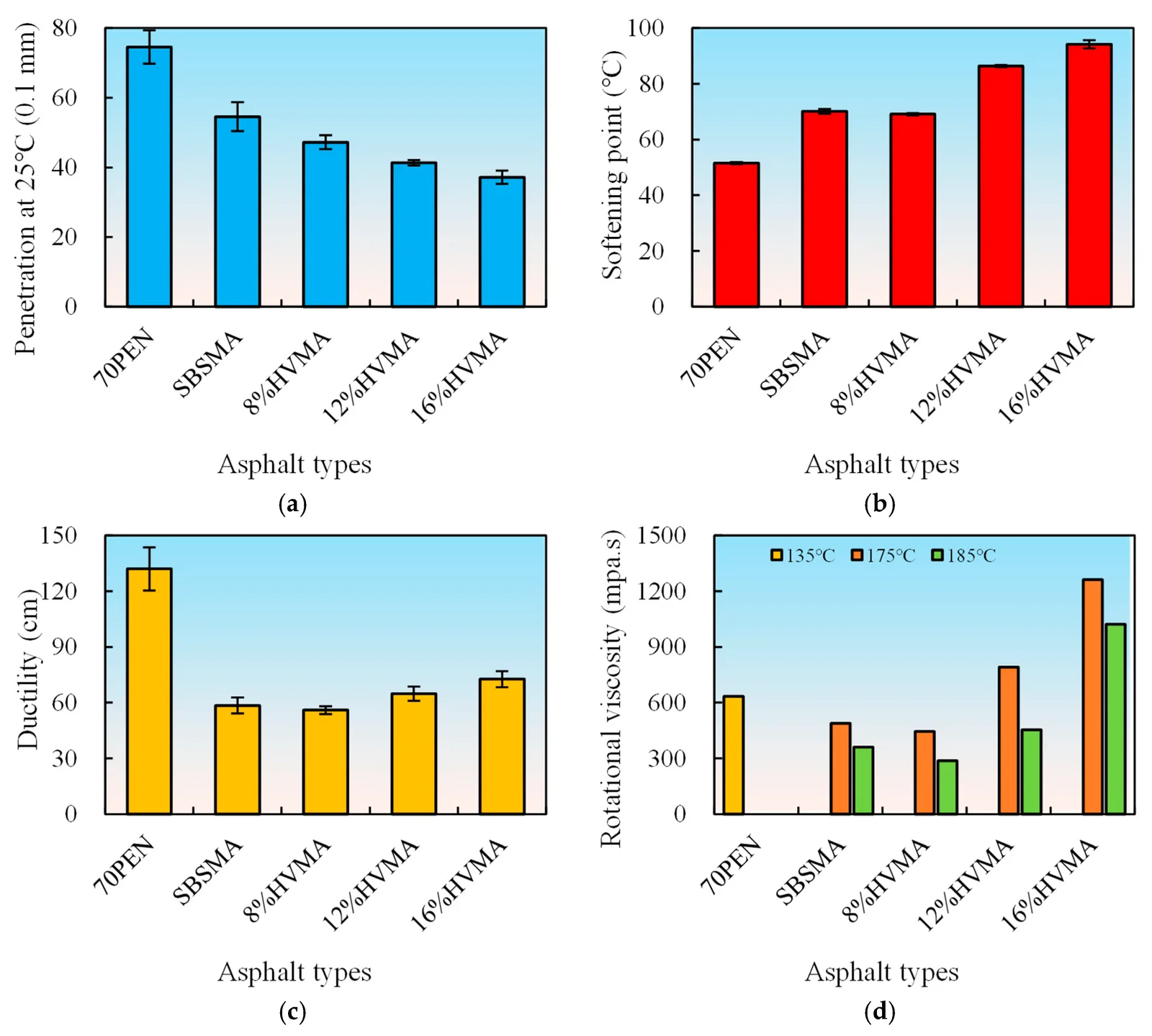
Basic physical properties of HVMA: (**a**) Penetration; (**b**) softening point; (**c**) ductility; (**d**) rotational viscosity. Error bars represent standard deviation (SD), *n* = 3.

The basic physical properties of HVMA with different high-viscosity modifier contents are presented in Figure 7. The corresponding data of 70PEN and SBSMA are also included for comparison. As shown in Figure 7, with increasing high-viscosity modifier content, the penetration of HVMA at 25 °C gradually decreases, whereas the softening point, low-temperature ductility, and rotational viscosity gradually increase. These results indicate that the incorporation of high-viscosity modifiers enhances the hardness, thermal stability, and low-temperature extensibility of HVMA, but simultaneously reduces its workability during construction.

In addition, the softening point and low-temperature ductility of 8%HVMA are comparable to those of SBSMA, while its penetration and rotational viscosity are slightly lower than those of SBSMA. According to CJJ/T 190-2012, the requirements for HVMA used in porous asphalt pavements specify that the penetration at 25 °C, softening point, and ductility at 5 °C should be no less than 40 (0.1 mm), 80 °C, and 30 cm, respectively. Therefore, the softening point of 8% HVMA is lower than 80 °C and does not satisfy the requirements for porous asphalt pavement applications, whereas 12% HVMA and 16% HVMA meet the corresponding performance requirements. From an engineering perspective, although further increasing the modifier dosage improves several rheological properties, the accompanying increase in viscosity may adversely affect mixing and compaction. Therefore, within the investigated dosage range, 12% HVMA appears to provide a more balanced compromise between performance enhancement and workability. Further mixture-level evaluation is still needed to determine the appropriate production temperature, mixing and compaction conditions, and economic feasibility in practical applications.

#### 3.2.2. Frequency and Temperature Sweep Test Results

**Figure 8:**
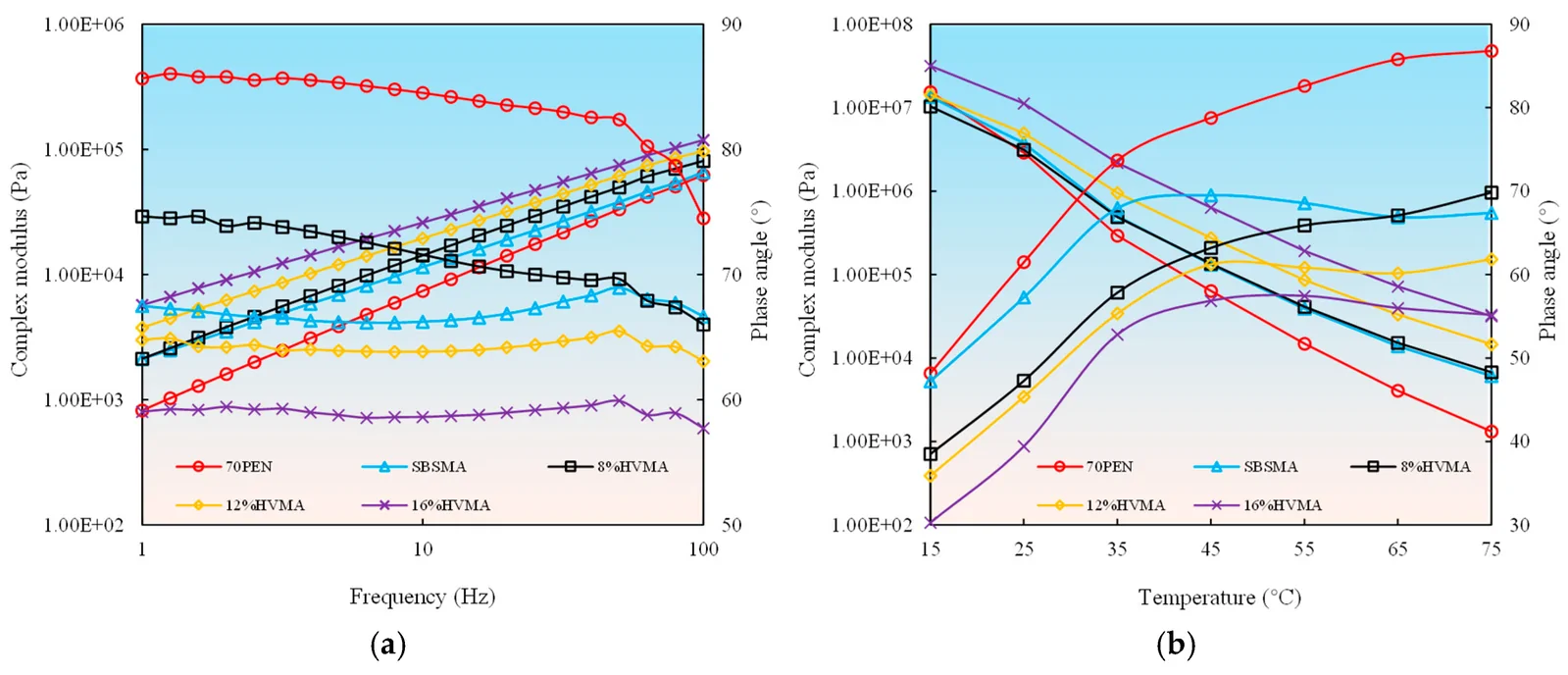
Frequency sweep and Temperature sweep test results of HVMA: (**a**) Frequency sweep; (**b**) temperature sweep. Data are expressed as mean values from two parallel replicates (*n* = 2).

The linear viscoelastic rheological properties of HVMA can be characterized by two parameters: complex modulus and phase angle. The complex modulus reflects the stiffness of HVMA, while the phase angle represents its viscoelastic behavior and the relative proportions of viscous and elastic components. The results of frequency sweep and temperature sweep tests for HVMA with different high-viscosity modifier contents are shown in Figure 8.

As shown in Figure 8a, the frequency sweep results indicate that the complex modulus of HVMA continuously increases with increasing loading frequency [40]. At all tested frequencies, 70PEN exhibits the lowest complex modulus. With increasing high-viscosity modifier content, the complex modulus of HVMA gradually increases, and the complex modulus of 8% HVMA is close to that of SBSMA. These results demonstrate that the incorporation of polymers increases the stiffness of asphalt binders. Within the loading frequency range of 1–100 Hz, 70PEN exhibits the highest phase angle, indicating a higher proportion of viscous components in 70PEN. With the addition of high-viscosity modifiers, the phase angle of HVMA gradually decreases, suggesting that polymer incorporation increases the elastic component while reducing the viscous component of asphalt binders. The phase angle of 8% HVMA is higher than that of SBSMA, whereas the phase angles of 12% HVMA and 16% HVMA are lower than that of SBSMA. This indicates that the elastic component of 8% HVMA is lower than that of SBSMA, while 12% HVMA and 16% HVMA exhibit higher elastic characteristics than SBSMA. The reduced frequency sensitivity with increasing modifier dosage is consistent with the behavior previously reported for highly polymer-modified asphalt [41], in which the elastic contribution associated with polymer-rich structures becomes increasingly pronounced as polymer content increases. However, the present frequency-sweep test does not directly characterize polymer-network morphology. Therefore, the reduced frequency dependence observed here should be interpreted as rheological evidence consistent with an enhanced polymer-related elastic contribution rather than direct proof of network formation.

As shown in Figure 8b, the temperature sweep results demonstrate that the complex modulus of HVMA continuously decreases, whereas the phase angle continuously increases with increasing temperature. At all test temperatures, 70PEN exhibits the lowest complex modulus, followed by SBSMA and 8% HVMA, while 12% HVMA and 16% HVMA show higher complex modulus values, with 16%HVMA exhibiting the highest stiffness. This further confirms that polymer incorporation enhances the stiffness of asphalt binders. The phase angle of 70PEN is the highest, and no obvious phase angle plateau region is observed over the entire temperature range of 15–75 °C. With increasing high-viscosity modifier content, the phase angle of HVMA decreases throughout the temperature range. In the temperature range of 15–45 °C, the phase-angle variation of HVMA followed a trend similar to that of 70PEN, suggesting that the rheological response within this temperature range remained strongly associated with the asphalt matrix.

At 45–75 °C, the temperature sensitivity of the phase angle gradually decreased with increasing modifier dosage. The phase-angle plateau was not pronounced for 8% HVMA, whereas distinct plateau regions were observed for 12% HVMA and 16% HVMA. Similar plateau behavior [42] has been reported for highly polymer-modified asphalt and has generally been associated with an increasing elastic contribution from polymer-related structures at elevated temperatures. As temperature increases, the viscosity of the asphalt matrix decreases, potentially making the rheological contribution associated with polymer-rich structures more pronounced. Nevertheless, the present rheological tests do not directly visualize polymer-network morphology or independently quantify the mechanical contributions of the asphalt and polymer phases. Therefore, the more pronounced phase-angle plateaus observed for 12% HVMA and 16% HVMA should be regarded as rheological evidence consistent with a stronger polymer-related elastic contribution rather than direct proof of a fully developed or dominant polymer network.

**Figure 9:**
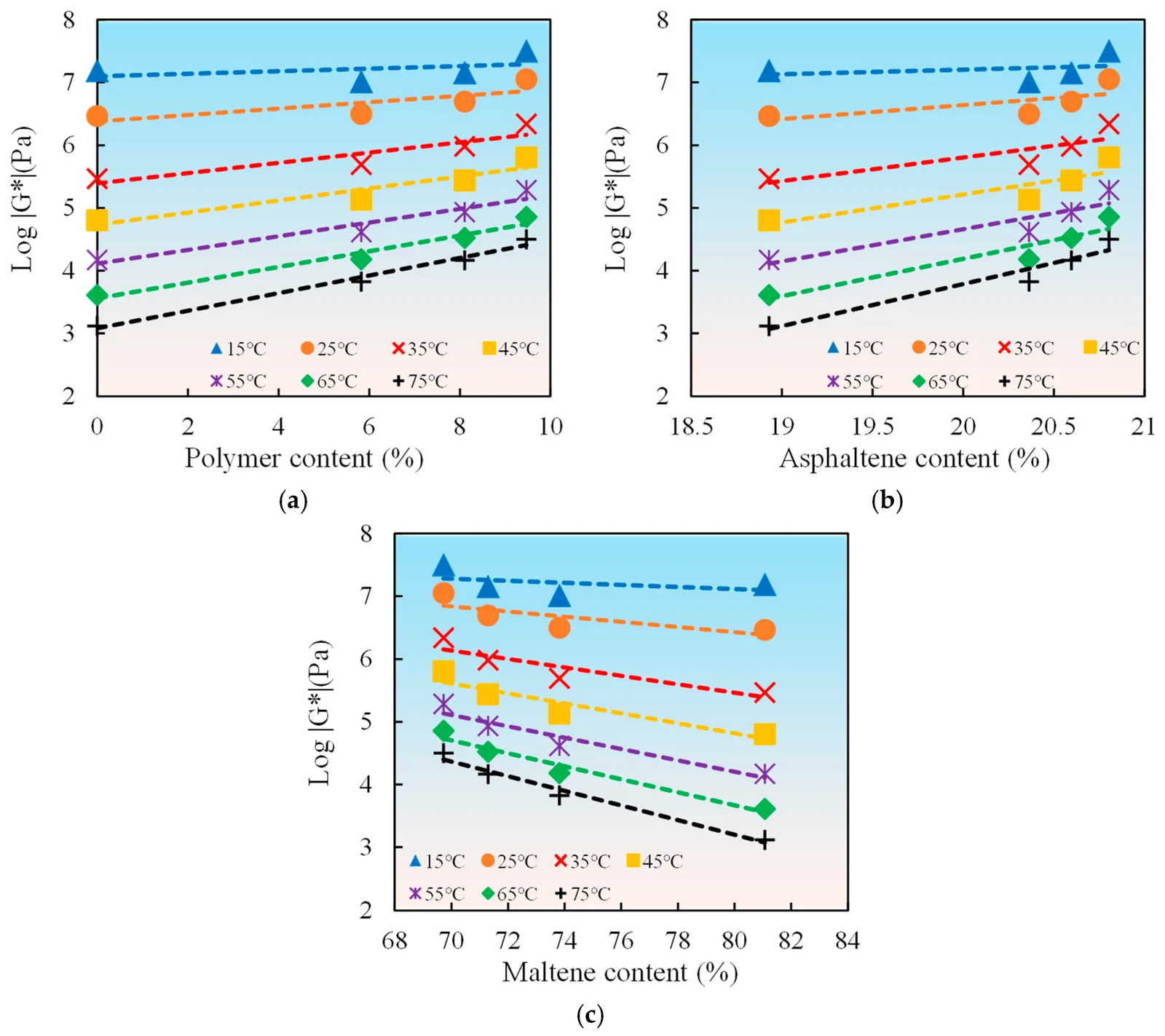
Effect of component composition on the complex modulus of HVMA: (**a**) log |G^*^| & polymer content; (**b**) log |G^*^| & asphaltene content; (**c**) log |G^*^| & maltene content.

**Figure 10:**
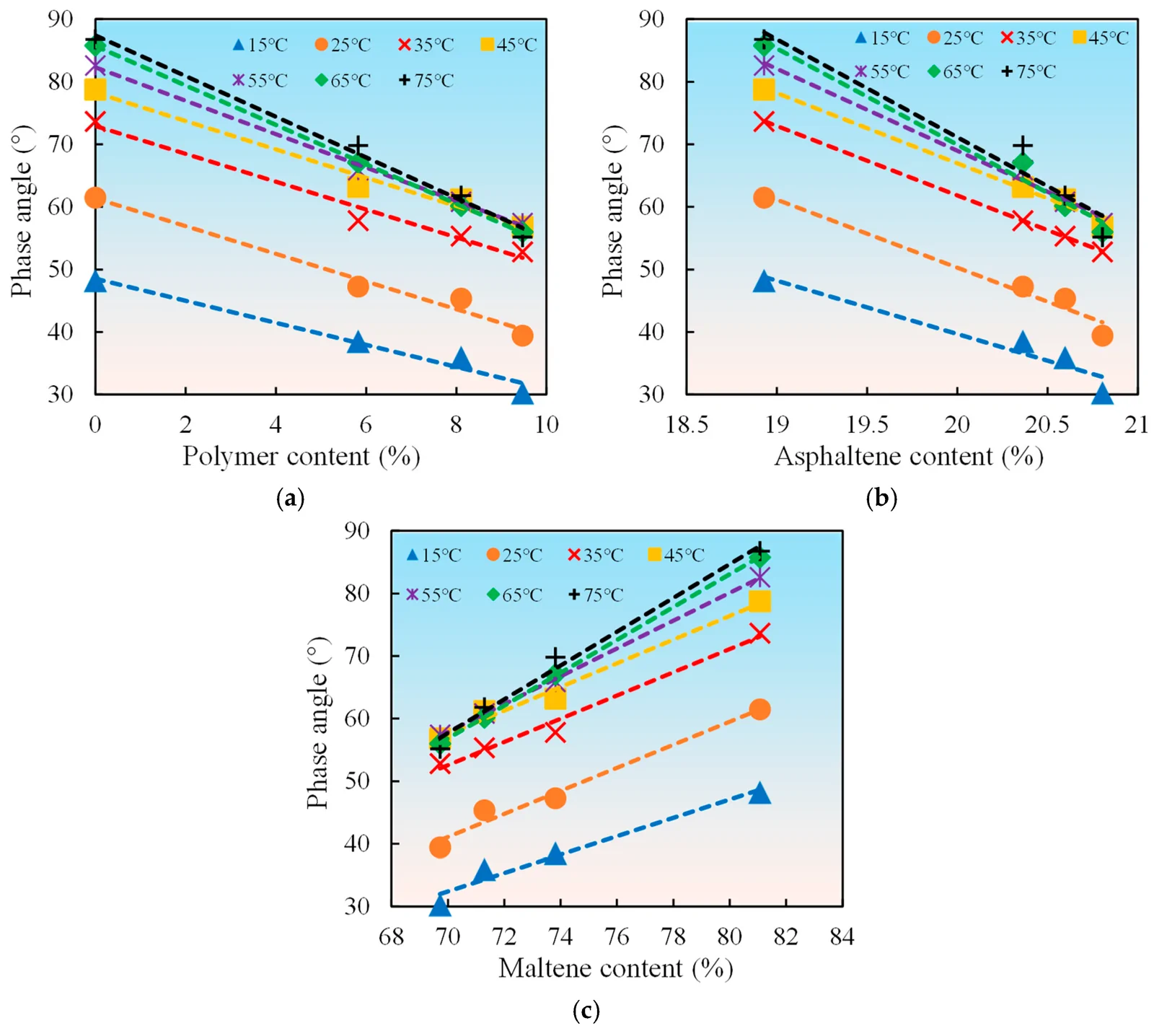
Effect of component composition on the phase angle of HVMA: (**a**) Phase angle & polymer content; (**b**) phase angle & asphaltene content; (**c**) phase angle & maltene content.

Figure 9 and Figure 10 illustrate the effects of HVMA component composition on its complex modulus and phase angle at different temperatures. It can be observed that the complex modulus of HVMA exhibits a positive correlation with the contents of polymer and asphaltene fractions, while showing a negative correlation with the maltene content. In addition, a higher proportion of asphaltene components in the asphalt phase leads to greater stiffness and modulus of HVMA. The phase angle of HVMA exhibits negative correlations with the polymer and asphaltene contents, but a positive correlation with the maltene content. These results suggest that the maltene fraction in the asphalt phase increases the viscous contribution of HVMA, thereby increasing its phase angle, whereas the polymer and asphaltene fractions reduce the viscous proportion and enhance the elastic response of HVMA. These associations are consistent with the generally higher elastic contribution associated with polymer-related structures and the greater molecular mobility associated with maltene-rich fractions. However, because these compositional indicators vary simultaneously with modifier dosage, the observed relationships should be interpreted as coupled associations rather than independent causal effects of individual fractions.

#### 3.2.3. Rheological Master Curves

**Figure 11:**
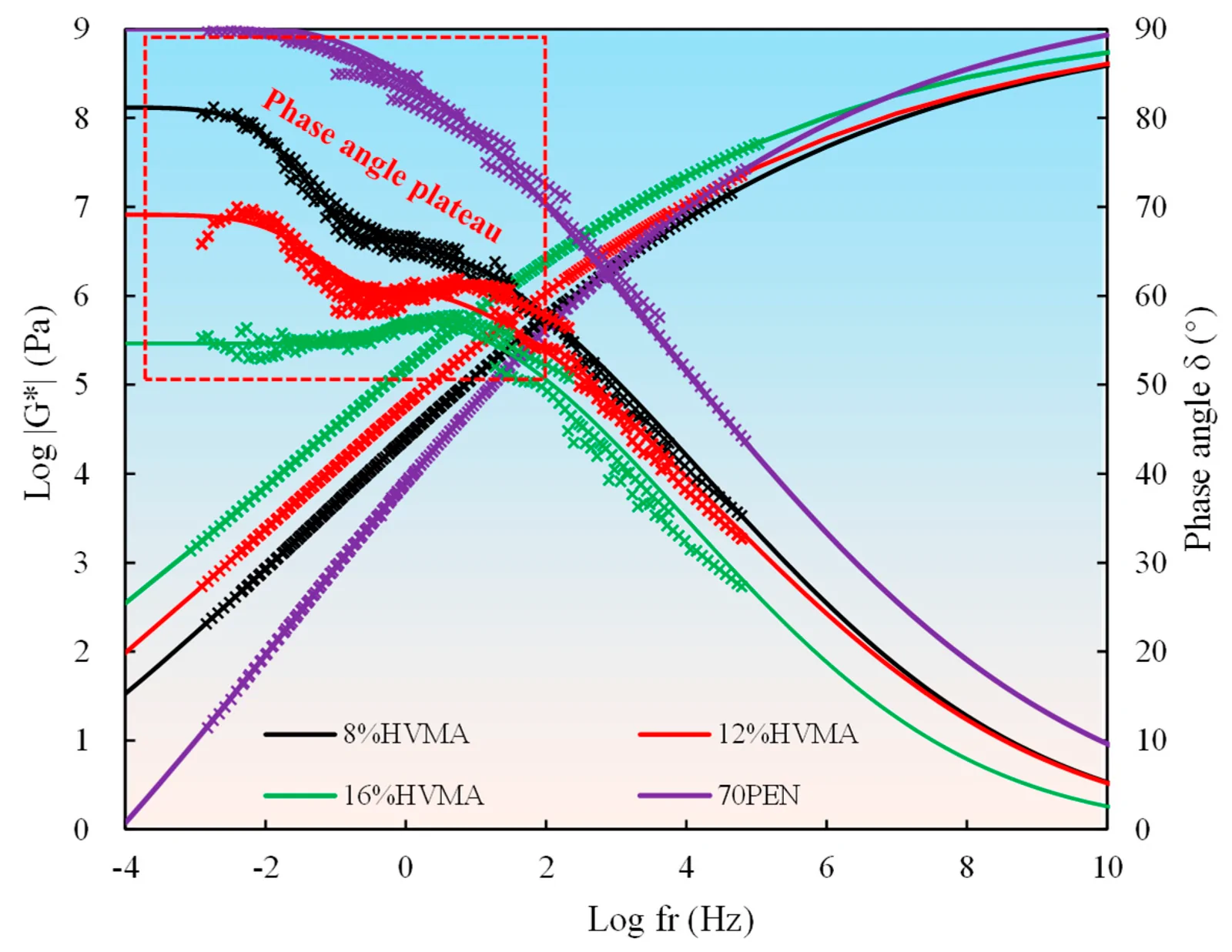
Rheological master curves of HVMA.

The rheological master curves of HVMA are presented in Figure 11. As shown in Figure 11, the complex modulus master curves of HVMA gradually shift upward with increasing high-viscosity modifier content. In the low-frequency region (corresponding to high temperatures), the complex modulus of HVMA is pronouncedly higher than that of 70PEN, whereas in the high-frequency region (corresponding to low temperatures), the complex modulus of HVMA is lower than that of 70PEN. These results indicate that the incorporation of polymers can pronouncedly enhance the stiffness and modulus of HVMA under high-temperature conditions. However, at low temperatures, the presence of polymer components reduces the stiffness and modulus of HVMA, thereby mitigating the risk of brittle failure caused by excessive hardening.

The phase angle master curves of HVMA exhibit marked differences compared with that of 70PEN. With the incorporation of high-viscosity modifiers, the phase angle master curves of HVMA gradually shift downward. In the intermediate- and low-frequency regions, HVMA exhibits an apparent phase angle plateau, which is absent in the phase angle master curve of 70PEN. According to previous studies [43], the formation of the phase angle plateau region in polymer-modified asphalt within the intermediate- and low-frequency ranges is mainly attributed to the elastic contribution of the polymer spatial network structure at high temperatures [44]. Similar phenomena have also been reported by other researchers [10]. In this study, the mechanism responsible for the formation of the phase angle plateau region in HVMA is further interpreted.

According to the time–temperature superposition principle, high frequencies in the rheological master curves are conventionally associated with low temperatures, whereas low frequencies are associated with high temperatures. In the high-frequency/low-temperature region, the rheological response of HVMA remains relatively close to the characteristic response of the asphalt matrix, suggesting a comparatively stronger contribution from the asphalt-related component under these conditions. As the reduced frequency decreases, the phase angle gradually departs from that of 70PEN and develops a plateau, indicating an increasingly evident elastic contribution associated with the polymer-related structure. However, the thermal stability of the asphalt phase is markedly lower than that of the polymer phase. With increasing temperature (i.e., in the intermediate-frequency and intermediate-temperature region, above the softening point), the viscosity of the asphalt phase gradually decreases, and its ability to constrain the polymer phase weakens. Consequently, the spatial network structure formed by the polymer phase may contribute more noticeably to the elastic response of HVMA. The polymer phase and asphalt phase jointly influence the rheological behavior of HVMA, with the polymer phase potentially becoming increasingly influential. This transition is manifested by the formation of a phase angle plateau region.

With further increasing temperature, the viscosity of the asphalt phase in HVMA decreases continuously. At this stage, the unrelaxed polymer molecules are dispersed within the relaxed asphalt phase, and the asphalt phase can no longer effectively restrict the influence of the polymer network structure on the overall rheological response. Therefore, the relative contribution of the polymer phase may increase, which may even result in a further decrease in phase angle (e.g., 12% HVMA).

These rheological trends are consistent with an increasing relative contribution of polymer-related structures as temperature increases and reduced frequency decreases. However, the master-curve analysis reflects the overall response of the composite binder and cannot independently quantify the mechanical contribution of each phase. Moreover, because the asphalt and polymer-related structures may exhibit different temperature sensitivities, highly polymer-modified asphalt can show thermorheologically complex behavior. Therefore, the present time–temperature superposition should be regarded as an empirical representation of the overall rheological response, and the inferred changes in phase contribution as mechanistic interpretations rather than direct evidence of phase dominance or strict thermorheological simplicity.

**Figure 12:**
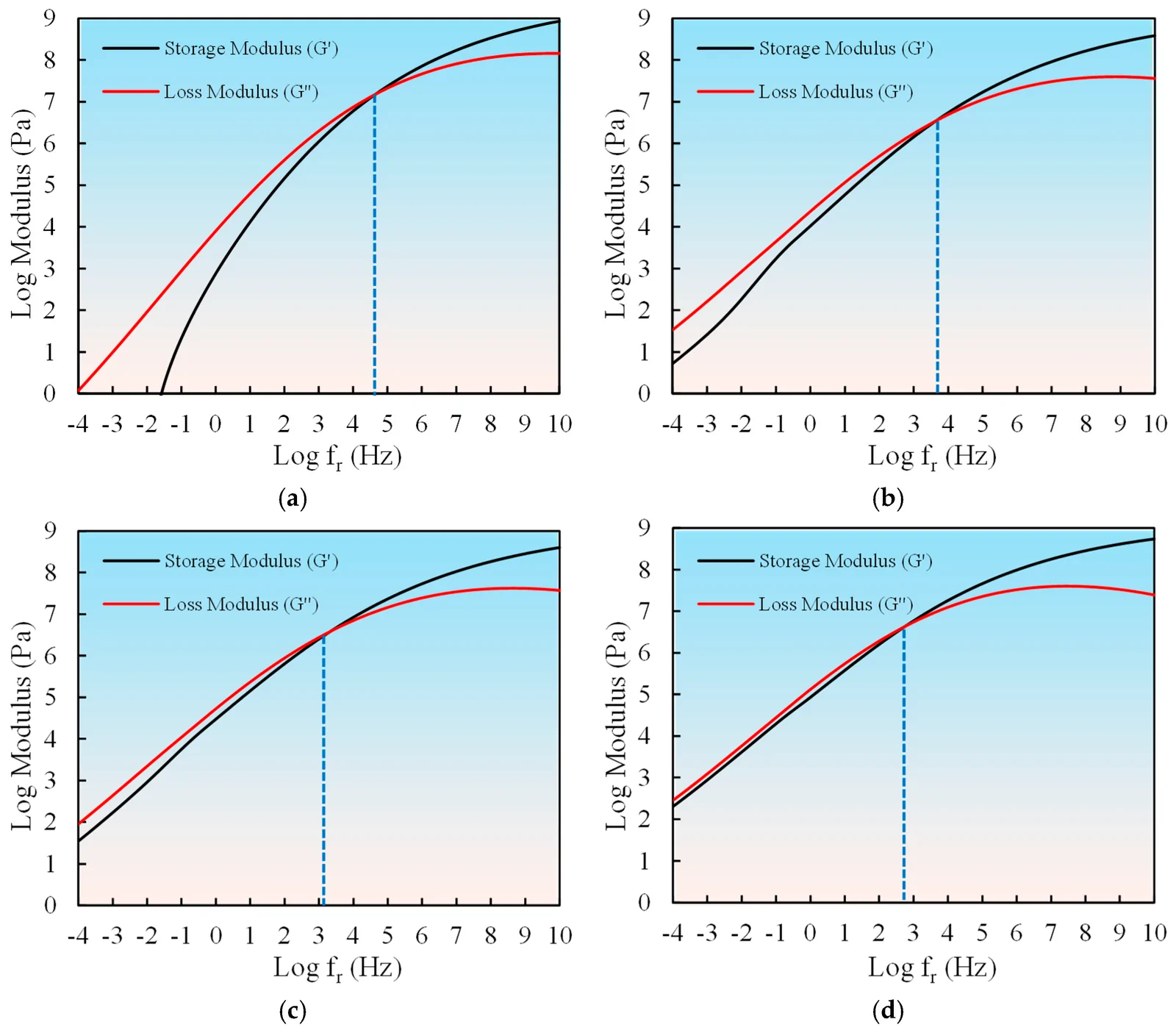
Viscoelastic transition frequency of HVMA: (**a**) 70PEN; (**b**) 8% HVMA; (**c**) 12% HVMA; (**d**) 16% HVMA. The vertical blue dashed line indicates the viscoelastic transition frequency (VTF).

**Figure 13:**
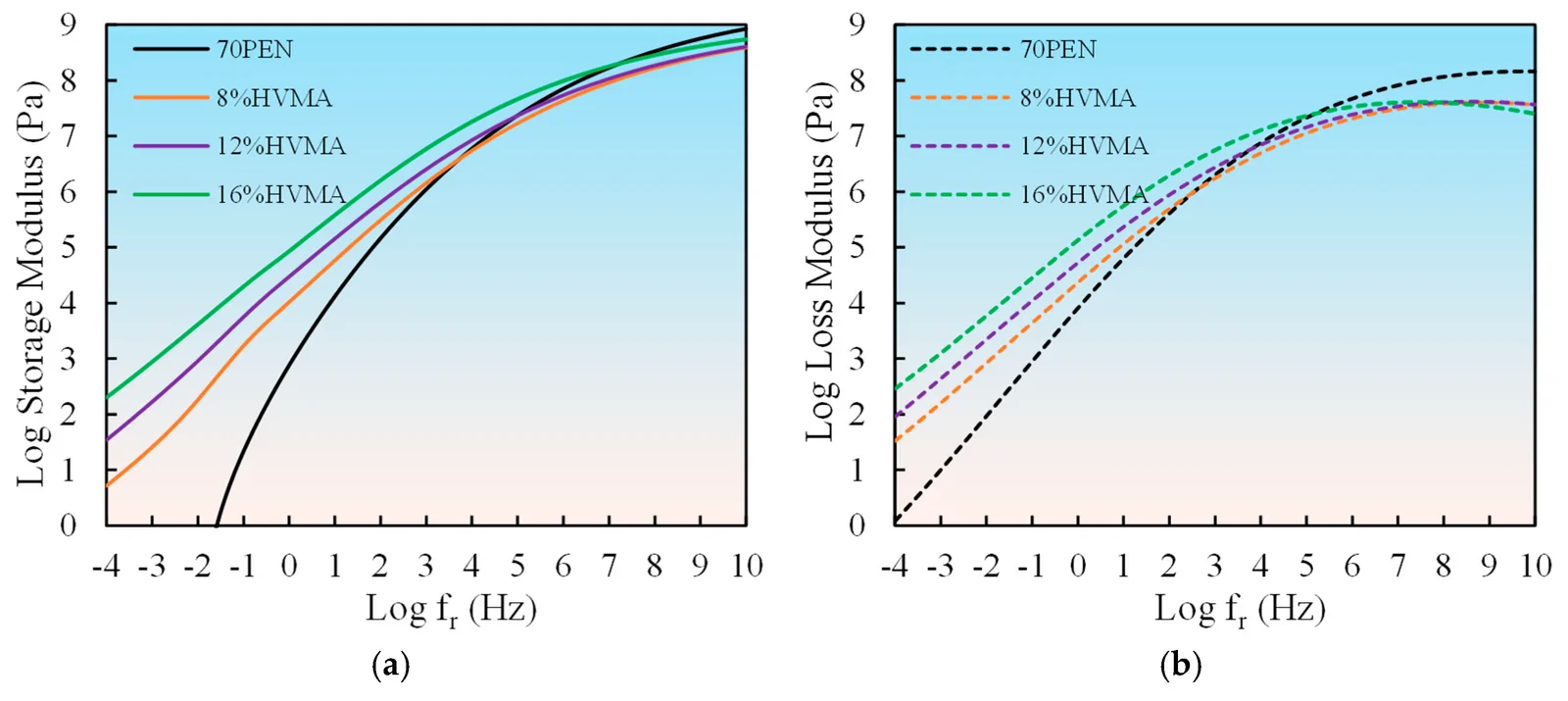
Comparison of storage modulus (*G*′) and loss modulus (*G*″) of HVMA: (**a**) Storage modulus (*G*′); (**b**) loss modulus (*G*″).

To further verify the aforementioned segmented dominance mechanism between the asphalt phase and polymer phase and the formation process of the phase angle plateau region in HVMA, the storage modulus (*G*′) and loss modulus (*G*″) master curves of HVMA were also constructed, as shown in Figure 12 and Figure 13. Three aspects are mainly discussed and analyzed as follows:

(1) Viscoelastic transition frequency (VTF): As shown in Figure 12, both the storage modulus (*G*′) and loss modulus (*G*″) of HVMA gradually increase with increasing reduced frequency, and an intersection point can be observed between the two curves. This intersection point is defined as the viscoelastic transition frequency (VTF) of HVMA [45]. Before the VTF point, the *G*′ value of HVMA is lower than the *G*″ value, indicating that HVMA exhibits predominantly viscous behavior. After the VTF point, the *G*′ value becomes higher than the *G*″ value, indicating that HVMA exhibits predominantly elastic behavior. The log(VTF) value of 70PEN is approximately 4.7. With increasing high-viscosity modifier content, the VTF point of HVMA gradually shifts toward lower frequencies. The log(VTF) values of 8% HVMA, 12% HVMA, and 16% HVMA are approximately 3.8, 3.2, and 2.8, respectively. These results indicate that polymer incorporation is associated with a shift in the viscoelastic transition toward lower frequencies, reflecting a more pronounced elastic response under the tested conditions. For 70PEN, the binder exhibits viscous behavior at high temperatures and elastic behavior at low temperatures. With increasing polymer content, the increasing contribution of the polymer phase may alter the overall rheological response of HVMA, potentially reflecting the development of polymer-related structures that enhance its elastic response. Consequently, the viscoelastic transition occurs at a higher frequency (corresponding to a lower temperature), indicating an earlier transition from viscous to elastic behavior during cooling.

(2) Comparison of *G*′ and *G*″ values of different asphalt binders at high and low frequency regions: According to Figure 13, marked differences in *G*′ values among different asphalt binders are observed in the low-frequency region. 70PEN exhibits a very low *G*′ value at low frequencies, indicating a highly viscous response and almost minimal elastic behavior under high-temperature conditions. With increasing high-viscosity modifier content, the *G*′ value of HVMA in the low-frequency region gradually increases, which is mainly attributed to the elastic contribution of the polymer phase at elevated temperatures. The dominant elastic response of the polymer phase at high temperatures is also responsible for the formation of the phase angle plateau region in HVMA. In contrast, the differences in *G*′ values among different asphalt binders in the high-frequency region are relatively small. This indicates that the polymer phase provides limited elastic contribution to HVMA at low temperatures, and the low-temperature rheological behavior of HVMA is mainly governed by the asphalt phase. The differences in *G*″ values among different asphalt binders in the low-frequency region are much smaller than those observed for *G*′, which is consistent with differences in the elastic response associated with polymer modification at high temperatures. In the high-frequency region, the *G*″ values of HVMA are lower than that of 70PEN. This difference may be attributed to changes in the overall viscoelastic response associated with polymer modification, although such contribution is largely masked by the elastic response of the asphalt phase.

(3) Comparison of lg *G*″ − lg *G*′ values of different asphalt binders at high and low frequency regions: Since lg *G*″ − lg *G*′ = lg(*G*″/*G*′), the parameter lg *G*″ − lg *G*′ can be used to characterize the ratio between viscous and elastic components of HVMA and is therefore defined as the viscoelastic ratio. The viscoelastic ratios (lg *G*″ − lg *G*′) of different asphalt binders at high frequency (10^10^ Hz) and low frequency (10^−4^ Hz) are summarized in Table 3. At low frequency and high temperature, the viscoelastic ratio of 70PEN reaches 7.57, indicating that the *G*″ value is much higher than the *G*′ value and that 70PEN exhibits an almost purely viscous behavior. With increasing high-viscosity modifier content, the viscoelastic ratio decreases pronouncedly. This is consistent with an increased elastic contribution associated with polymer modification, which increases *G*′ relative to *G*″ and accompanies the development of the phase angle plateau region. At high frequency and low temperature, the difference in viscoelastic ratio among different asphalt binders becomes pronouncedly smaller. This indicates that the low-temperature rheological behavior of HVMA shows smaller differences in the relative contributions of viscous and elastic responses among the tested binders, while the influence of the polymer phase is relatively limited under such conditions.

#### 3.2.4. Viscoelastic Transition Temperature (VTT)

**Figure 14:**
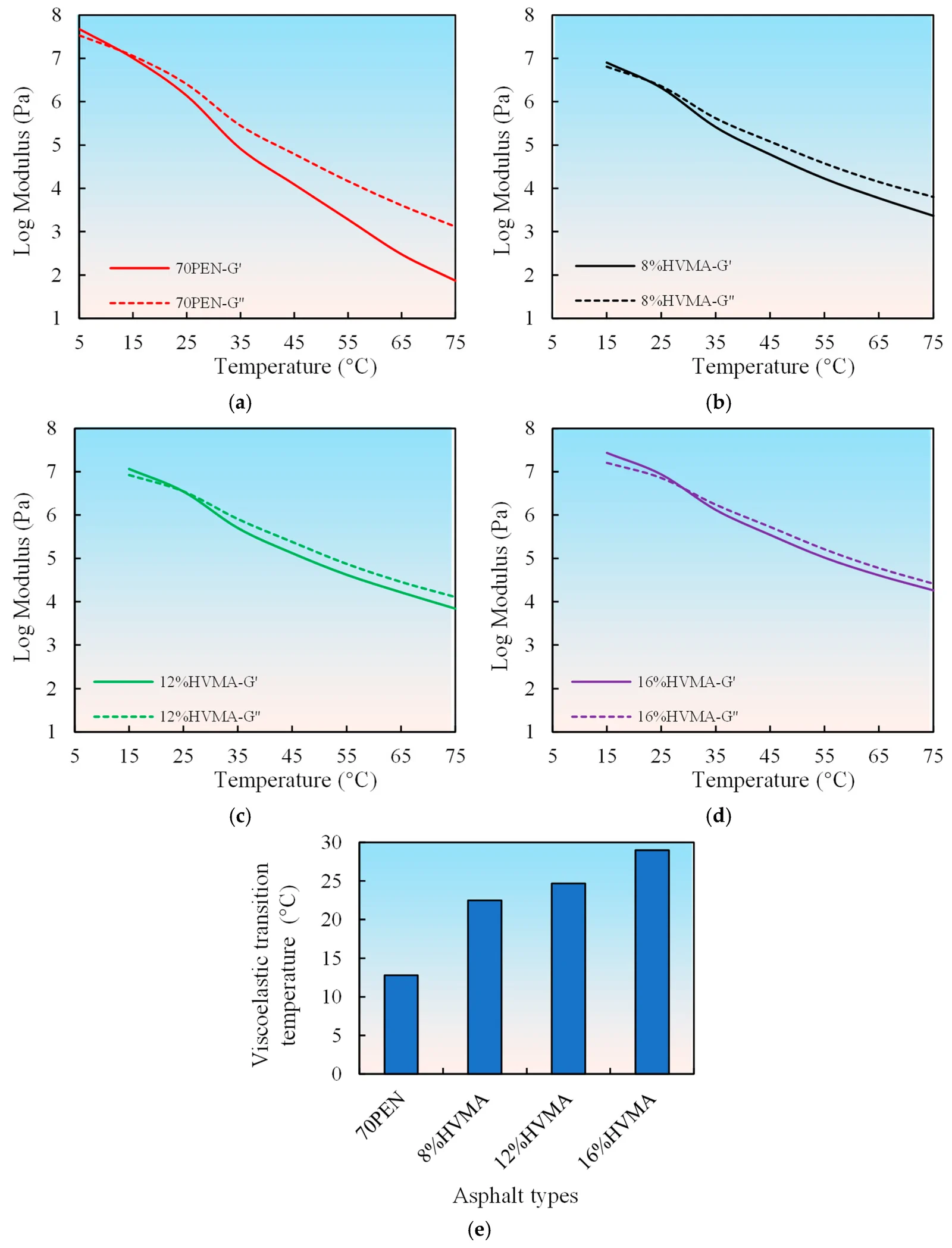
Viscoelastic transition temperature of HVMA: (**a**) 70PEN; (**b**) 8% HVMA; (**c**) 12% HVMA; (**d**) 16% HVMA; (**e**) VTT.

Figure 14 summarizes the viscoelastic transition temperature (VTT) results of 70PEN and HVMA. As shown in Figure 14a–d, the *G*′ and *G*″ values of both 70PEN and HVMA gradually decrease with increasing temperature, and an intersection point is observed between the two curves. This intersection point is defined as the viscoelastic transition temperature (VTT).

Below the VTT, the *G*′ value of HVMA is greater than the *G*″ value, indicating predominantly elastic behavior. Above the VTT, the *G*′ value becomes lower than the *G*″ value, indicating predominantly viscous behavior. With increasing high-viscosity modifier content, the difference between *G*′ and *G*″ at high temperatures gradually decreases, further demonstrating the dominant role of the polymer phase in governing the high-temperature rheological behavior of HVMA.

As shown in Figure 14e, the VTT of HVMA gradually increases with increasing high-viscosity modifier content. On the one hand, the elastic structure of the polymer phase provides an additional elastic contribution to the asphalt phase, thereby delaying the overall transition of HVMA from an elasticity-dominated state to a viscosity-dominated state as the temperature increases. On the other hand, the polymer absorbs part of the light components in the asphalt and undergoes swelling. Consequently, the relative content of light components decreases, whereas that of heavy components increases, which also promotes a partial shift in the rheological contribution of HVMA from viscous to elastic behavior.

**Figure 15:**
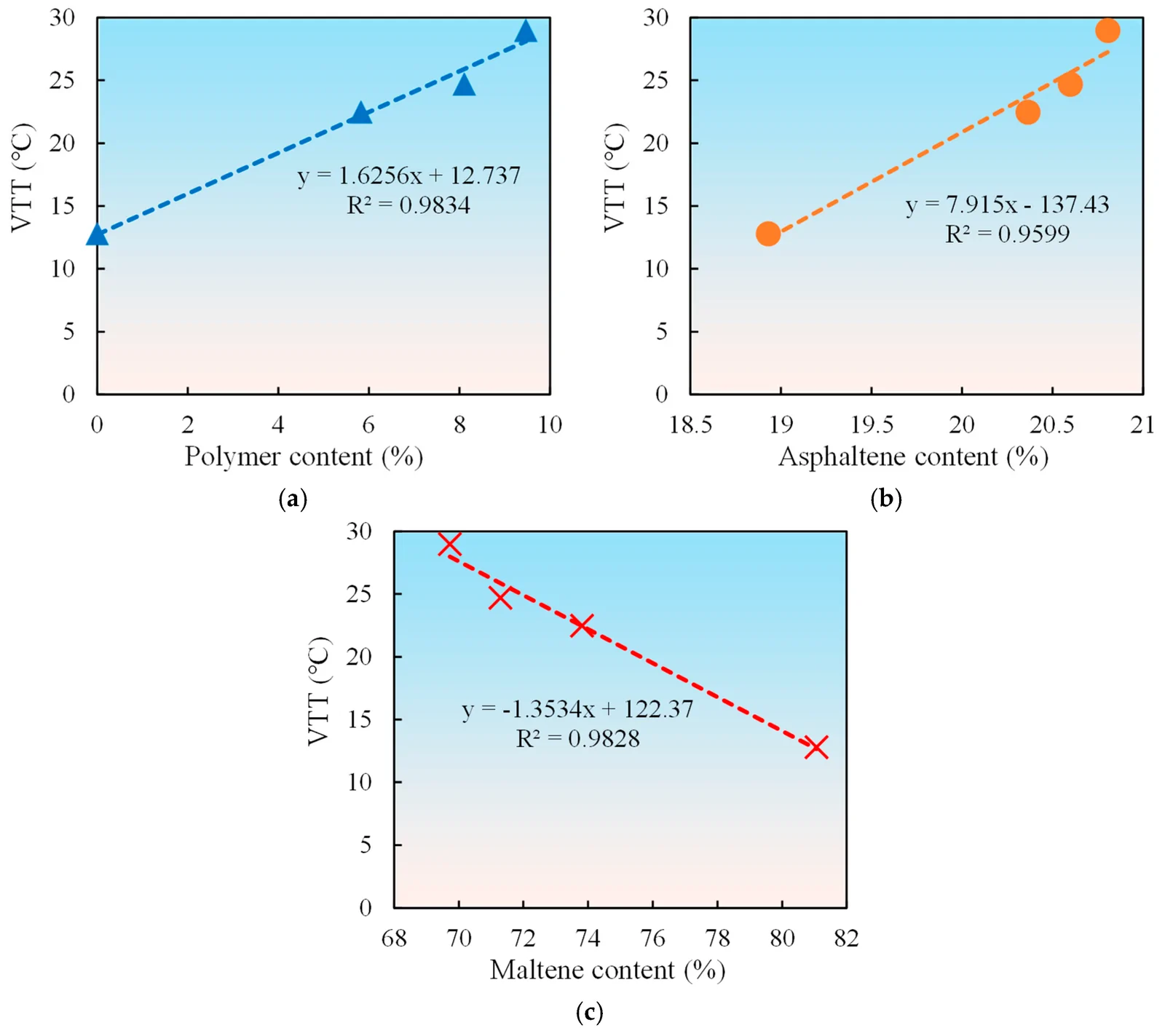
Effect of component composition on the VTT values of HVMA: (**a**) VTT & polymer content; (**b**) VTT & asphaltene content; (**c**) VTT & maltene content.

Figure 15 illustrates the relationships between the VTT of HVMA and its component composition and phase structure. As shown in Figure 15, the VTT of HVMA is positively correlated with the polymer and asphaltene contents but negatively correlated with the maltene content. The relative proportions of asphaltenes and maltenes in the asphalt phase affect the balance between the elastic and viscous contributions of HVMA and consequently influence its VTT. As the asphaltene content increases and the maltene content decreases, the elastic contribution of HVMA increases, whereas its viscous contribution decreases, resulting in a higher VTT. In contrast, polymer molecules influence the VTT of HVMA indirectly. Specifically, polymer molecules absorb part of the light components and undergo swelling, thereby altering the relative composition of the asphalt phase and ultimately affecting the VTT of HVMA.

#### 3.2.5. Multiple Stress Creep Recovery (MSCR) Test

**Figure 16:**
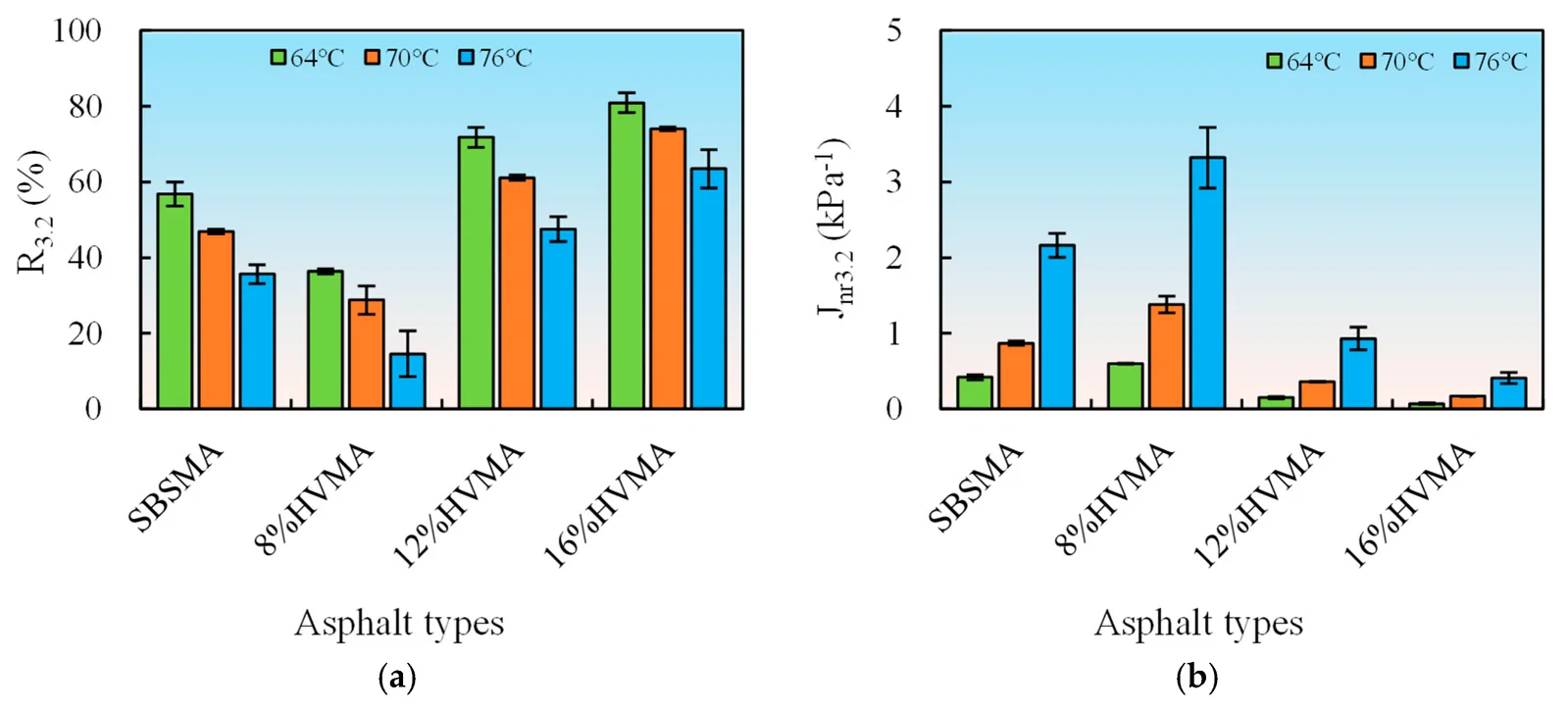
MSCR results of HVMA: (**a**) *R*_3.2_; (**b**) *J*_nr,3.2_. Data are expressed as mean values with standard deviation (error bars) from two parallel replicates (*n* = 2).

The MSCR test provides two key parameters: percent recovery and non-recoverable creep compliance [46]. Percent recovery can be used to characterize the contribution of the polymer phase in polymer-modified asphalt, whereas non-recoverable creep compliance exhibits a strong correlation with field rutting performance and can therefore be used to evaluate the high-temperature resistance of polymer-modified asphalt to permanent deformation [47]. The percent recovery (*R*_3.2_) and non-recoverable creep compliance (*J*_nr,3.2_) of HVMA at a stress level of 3.2 kPa are presented in Figure 16.

As shown in Figure 16, *R*_3.2_ increases and *J*_nr,3.2_ decreases with increasing high-viscosity modifier content [48]. This trend is consistent with the results reported by Zhou et al. [36], who found that the incorporation of SBS improved the high-temperature rheological performance and rutting resistance of high-viscosity modified asphalt. The incorporation of the high-viscosity modifier may promote the development of polymer-related structures within HVMA, which could contribute to enhancing its elastic recovery capacity and resistance to high-temperature rutting deformation. The *R*_3.2_ and *J*_nr,3.2_ values of SBSMA fall between those of 8% HVMA and 12% HVMA. This indicates that 8% HVMA exhibits lower elastic recovery capacity and poorer resistance to high-temperature permanent deformation than SBSMA, whereas 12% HVMA and 16% HVMA show substantially superior elastic recovery and rutting resistance compared with SBSMA.

**Figure 17:**
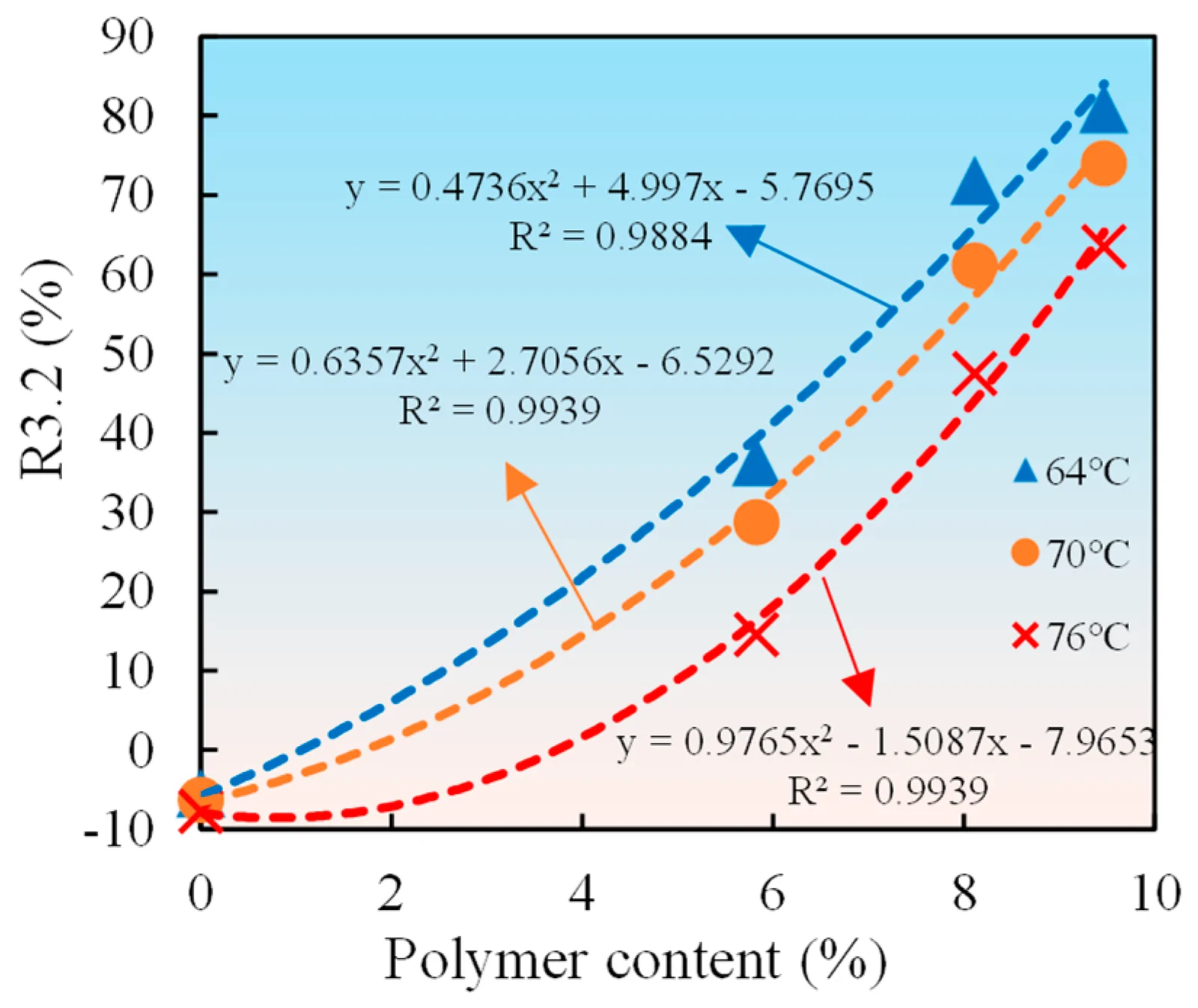
Effect of polymer phase on the elastic recovery ability of HVMA.

**Figure 18:**
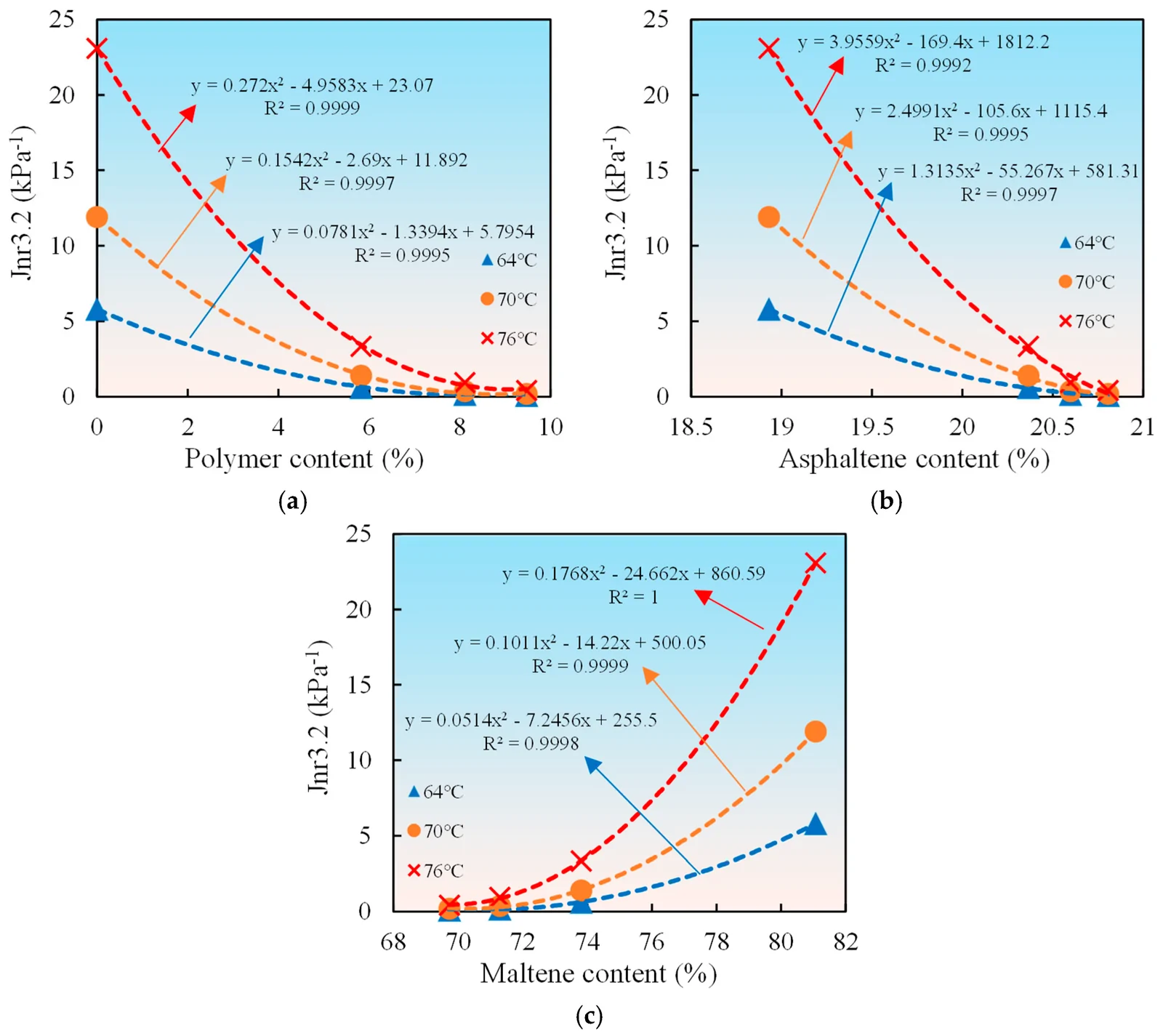
Effect of component composition on the high-temperature permanent deformation resistance of HVMA: (**a**) *J*_nr,3.2_ & polymer content; (**b**) *J*_nr,3.2_ & asphaltene content; (**c**) *J*_nr,3.2_ & maltene content.

The *R*_3.2_ value of 70PEN is negative, whereas those of HVMA are positive. The positive association between polymer content and elastic recovery suggests that polymer-related characteristics are closely associated with the elastic recovery behavior of HVMA. Therefore, Figure 17 only presents the effect of polymer content on the elastic recovery capacity (*R*_3.2_) of HVMA. A strong positive correlation is observed between the *R*_3.2_ value and polymer content. Figure 18 further illustrates the effects of HVMA component composition on its resistance to high-temperature permanent deformation, as characterized by *J*_nr,3.2_. The *J*_nr,3.2_ value is negatively correlated with the polymer and asphaltene contents but positively correlated with the maltene content. These results suggest that higher polymer and asphaltene contents are associated with lower non-recoverable creep compliance, whereas higher maltene content is associated with higher non-recoverable creep compliance, indicating corresponding associations with high-temperature deformation resistance.

### 3.3. Grey Relational Analysis Between Macroscopic and Microscopic Properties of HVMA

In this section, grey relational theory was employed to analyze the relationships between the macroscopic performance and microscopic properties of HVMA. Based on the preceding results, the macroscopic performance indicators and microscopic property indicators selected for HVMA are summarized in Table 4.

**Figure 19:**
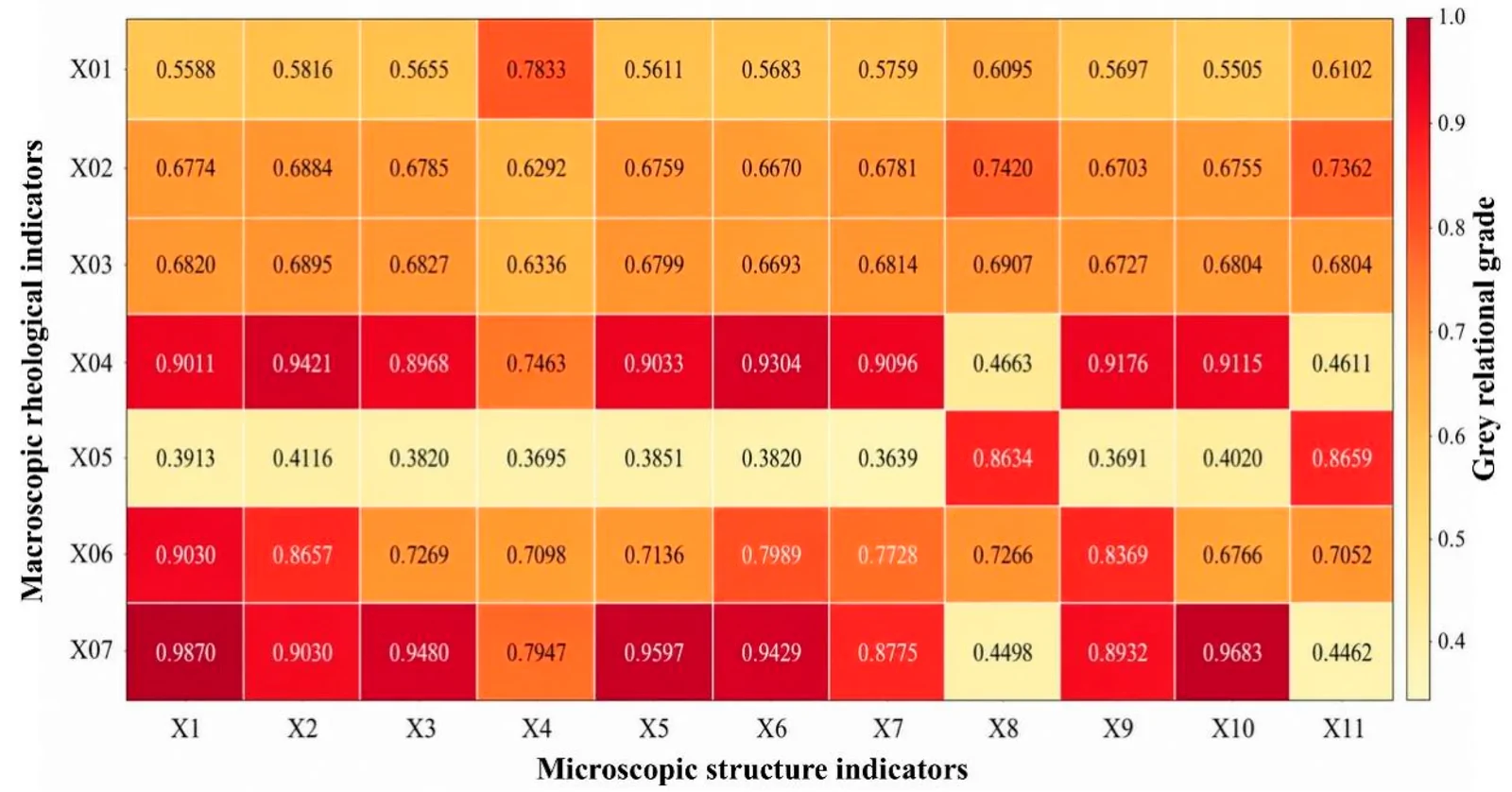
Grey relational analysis results between macroscopic and microscopic performance indicators of HVMAs.

The grey relational grades between the different macroscopic performance indicators of HVMA and all microscopic indicators are presented in Figure 19. The results demonstrate that the microscopic indicators showing relatively strong associations differ markedly among the various macroscopic performance indicators, reflecting distinct multiscale structure–property relationships in HVMA.

For the viscoelastic transition temperature (VTT), relatively high grey relational grades are observed with the PB and PS peak areas, polymer content, asphaltene content, polydispersity index, and weight-average molecular weight. This indicates that the VTT of HVMA is closely associated with the structural evolution of the SBS polymer, variations in the asphaltene fraction, and changes in the molecular weight distribution. With increasing high-viscosity modifier content, the number of SBS polymer chain segments gradually increases. The flexible PB segments and rigid PS segments may contribute to the formation of a spatial polymer network, thereby increasing the internal frictional resistance of the system. Meanwhile, the increase in asphaltene content and the shift of the molecular weight distribution toward higher values may further contribute to the stability of the colloidal structure and reduced molecular-chain mobility, which could explain the increased viscosity of the asphalt system. Therefore, the variation in VTT may be related to the combined contributions of SBS polymer-network reinforcement, asphaltene structural reorganization, and molecular-chain entanglement.

For the MSCR parameter *R*_3.2_, relatively high grey relational grades are observed with the PB and PS peak areas and polymer content, indicating that the high-temperature elastic recovery capacity is more strongly associated with SBS-related structural characteristics. As the polymer content increases, the flexible PB segments and rigid PS segments of SBS may contribute to the formation of a spatially crosslinked network. The relatively high relational grades of the PB and PS peak areas with rheological parameters are consistent with the polymer-network-related mechanisms proposed in previous studies [49]. In particular, chain entanglement and physical crosslinking have been widely used to explain the enhanced elastic response of SBS-modified asphalt; however, these structural processes were not directly measured in the present study. Therefore, the variation in *R*_3.2_ may be associated with the evolution of the SBS chain-segment structure and the potential development of an elastic polymer network in asphalt. In contrast, *J*_nr,3.2_ exhibits the highest grey relational grade with the maltene content, suggesting a stronger association between non-recoverable creep behavior and maltene-related characteristics. An increase in maltene content may reduce the constraining effect of the colloidal structure and promotes molecular-chain migration and rearrangement, potentially contributing to the accumulation of permanent deformation.

The complex shear modulus, |G^*^|, at different temperatures also exhibits pronounced temperature-dependent relationships with the microscopic structural parameters. At low temperature (15 °C), |G^*^| shows relatively high grey relational grades with the number-average molecular weight and maltene content, whereas at an intermediate temperature (45 °C), |G^*^| is mainly associated with the maltene fraction. These results indicate that the microscopic indicators most strongly associated with the complex shear modulus vary with temperature. At low temperatures, molecular-chain mobility is restricted, and the stiffness of the system may be influenced by molecular-chain length and intermolecular interactions. A higher number-average molecular weight may contribute to greater molecular-chain entanglement and improves the resistance of asphalt to external deformation. Meanwhile, the maltene fraction may also be relevant to the low-temperature viscoelastic behavior. As the temperature increases, molecular mobility is enhanced, potentially altering the relative contributions of different molecular characteristics to the viscoelastic response. The stronger association with maltene content at intermediate temperatures may be related to the flow characteristics of the maltene fraction. At high temperature (75 °C), several polymer- and asphalt-related indicators exhibit relatively high grey relational grades, suggesting that the high-temperature rheological behavior is associated with both polymer- and asphalt-related characteristics.

The phase angle plateau region, an important parameter for evaluating the viscoelastic balance of the material, exhibits relatively high grey relational grades with the PB and PS peak areas and polymer content. This indicates that the viscoelastic transition behavior of HVMA is strongly associated with the structural characteristics of the SBS polymer and its network-forming capability. The PB and PS peaks respectively reflect structural variations in the polybutadiene soft segments and polystyrene hard segments of SBS. Their high relational grades suggest a close relationship between the chain-segment composition and microstructural evolution of SBS play important roles in determining the viscoelastic balance of the material. With increasing polymer content, SBS molecular chains gradually form a more developed spatial network within the asphalt system. The flexible PB segments enhance molecular-chain entanglement, whereas the PS hard segments improve structural stability through physical crosslinking. These structural changes could contribute to a stronger elastic response and shifts the phase angle plateau toward lower values. Therefore, the evolution of the phase angle plateau region may be associated with SBS chain-segment structure and polymer-network reinforcement, consistent with their potential roles in the viscoelastic behavior of asphalt.

In summary, the grey relational analysis reveals the multiscale structure–property relationships among the chemical composition, molecular weight distribution, and rheological performance of HVMA. Different rheological indicators exhibited different degrees of association with the investigated microscopic characteristics. Specifically, VTT showed relatively high grey relational grades with SBS-related structural indicators, asphaltene-related characteristics, and molecular-weight parameters. *R*_3.2_ and the phase-angle plateau showed stronger associations with polymer-related indicators. In contrast, *J*_nr,3.2_ and |G^*^| at low and intermediate temperatures exhibited relatively strong associations with maltene-related characteristics, whereas |G^*^| at high temperatures showed relatively high relational grades with both polymer- and asphalt-related parameters. Overall, the enhanced performance of HVMA is accompanied by changes in SBS chain-segment regulation, molecular weight growth, and optimization of the asphalt colloidal structure. These observed associations are consistent with the potential contributions of polymer-network development and colloidal structural evolution to rheological performance, although their causal roles cannot be established from GRA alone. Therefore, the GRA results are interpreted primarily in terms of relative association trends, and small differences in relational grades should not be overinterpreted given the limited number of modifier levels.

Several limitations of the present study should be acknowledged. First, only three high-viscosity modifier dosages (8%, 12%, and 16%) were investigated, which limits the resolution for identifying possible dosage thresholds. Second, the polymer-, asphaltene-, and maltene-related fractions derived from GPC are operational classifications based on apparent molecular-weight ranges, and the calculated proportions may be affected by peak overlap and the adopted deconvolution procedure. Third, grey relational analysis evaluates the similarity of variation trends and therefore reflects association rather than causality; its rankings should be interpreted cautiously given the limited number of dosage levels. Finally, only unaged binders were evaluated in this study, and aging-induced changes in polymer integrity, molecular-weight distribution, and rheological behavior were not considered. Future work should include additional modifier levels, further validation of GPC-based component analysis, and RTFOT/PAV aging to examine the robustness and aging dependence of the identified structure–property relationships.

## 4. Conclusions and Recommendations

This study investigated high-viscosity modified asphalt (HVMA) prepared with different dosages of a high-viscosity modifier. A series of tests, including conventional physical property tests, gel permeation chromatography, Fourier transform infrared spectroscopy, frequency sweep, temperature sweep, rheological master-curve analysis, and multiple stress creep recovery testing, were conducted to systematically evaluate the effects of modifier dosage on the chemical composition, molecular weight distribution, component composition, and rheological properties of HVMA. On this basis, grey relational analysis was further employed to reveal the multiscale structure–property relationships between the microscopic composition and macroscopic rheological performance of HVMA. The main conclusions are as follows:

1. With increasing modifier dosage, HVMA exhibited lower penetration and non-recoverable creep compliance, but higher softening point, ductility, viscosity, complex modulus, percent recovery, and viscoelastic transition temperature, indicating improved high-temperature stability and elastic recovery at the expense of workability. Both 12% HVMA and 16% HVMA met the relevant requirements for porous asphalt pavements; however, 12% HVMA provided a more balanced compromise between performance and viscosity within the investigated range.
2. The high-viscosity modifier altered the apparent molecular-weight distribution of HVMA. With increasing modifier dosage, the weight-average molecular weight and the polymer- and asphaltene-related molecular-weight fractions increased, whereas the maltene-related fraction decreased. These changes may reflect the redistribution of asphalt components associated with polymer modification and the contribution of polymer species within overlapping apparent molecular-weight ranges. Therefore, the increase in the asphaltene-related fraction should be interpreted as a GPC-based molecular-weight classification rather than direct evidence of an increase in chemically defined asphaltenes.
3. HVMA exhibited a clear temperature- and frequency-dependent change in rheological response. Under low-temperature and high-frequency conditions, the response was more strongly associated with the asphalt matrix. With increasing temperature and decreasing reduced frequency, the polymer-related elastic contribution became increasingly evident, as indicated by the development of a phase-angle plateau, the increase in low-frequency storage modulus, and the decrease in the viscoelastic ratio. Compared with 8% HVMA, the more pronounced plateau characteristics of 12% HVMA and 16% HVMA are consistent with a stronger contribution from polymer-related structures.
4. Grey relational analysis revealed different degrees of association between the investigated microscopic characteristics and rheological parameters. VTT showed relatively high relational grades with SBS-related structural indicators, asphaltene-related characteristics, and molecular-weight parameters. *R*_3.2_ and the phase-angle plateau were more strongly associated with polymer-related indicators, whereas *J*_nr,3.2_ and |G^*^| at low and intermediate temperatures showed stronger associations with maltene-related characteristics. At high temperatures, |G^*^| exhibited relatively high relational grades with both polymer- and asphalt-related parameters. These GRA results represent statistical associations between the variation trends of microscopic and rheological parameters and should not be interpreted as direct causal relationships.

Several limitations should be acknowledged. Only three modifier dosages were investigated, and the GPC-derived component fractions may be affected by peak overlap and deconvolution uncertainty. In addition, GRA reflects association rather than causality, and only unaged binders were evaluated. Future work should include more dosage levels, improved validation of GPC-based component analysis, and RTFOT/PAV aging to further verify the robustness and aging dependence of the identified structure–property relationships.

## Figures and Tables

**Table 1:** Molecular weight characteristic parameters of HVMA.

Asphalts	Weight-Average Molecular Weight (*M*_w_)	Number-Average Molecular Weight (*M*_n_)	Polydispersity Index (PDI)
70PEN	1863.80	344.76	5.41
SBSMA	7620.59	339.45	22.45
8% HVMA	10,498.46	388.56	27.02
12% HVMA	12,428.32	378.51	32.84
16% HVMA	15,109.94	378.78	39.89

**Table 2:** Percentage contents of HVMA components based on the peak deconvolution fitting method.

Asphalts	Polymer (%)	Asphaltene (%)	Maltene (%)
70PEN	0.00	8.86	91.14
8% HVMA	5.97	10.56	83.47
12% HVMA	7.74	11.14	81.13
16% HVMA	8.52	12.01	79.48

**Table 3:** Viscoelastic parameter (lg *G*″ − lg *G*′) of HVMA.

Asphalts	70PEN	8% HVMA	12% HVMA
High-frequency region (10^10^ Hz)	7.57	0.81	0.42
Low-frequency region (10^−4^ Hz)	−0.77	−1.03	−1.04

**Table 4:** Performance indicators of HVMAs used for grey relational analysis.

Analysis Scale	Test Method	Parameters
Macroscopic scale	DSR	|G^*^|_15°C_ (*X*_01_), |G^*^|_45°C_(*X*_02_), |G^*^|_75°C_ (*X*_03_)
	MSCR	*R*_3.2_ (*X*_04_), *J*_nr,3.2_ (*X*_05_)
	Master curve	Phase angle plateau region (*X*_06_)
	VTT	VTT (*X*_07_)
Microscopic scale	FTIR	PB peak area (*X*_1_), PS peak area (*X*_2_)
	GPC	Weight-average molecular weight (*X*_3_), number-average molecular weight (*X*_4_), polydispersity index (*X*_5_), polymer content based on molecular weight distribution(*X*_6_), asphaltene content based on molecular weight distribution (*X*_7_), maltene content based on molecular weight distribution (*X*_8_), polymer content obtained by peak deconvolution (*X*_9_), asphaltene content obtained by peak deconvolution (*X*_10_), maltene content obtained by peak deconvolution (*X*_11_)

## Data Availability

The original contributions presented in this study are included in the article. Further inquiries can be directed to the corresponding author.
